# Context influences on TALE–DNA binding revealed by quantitative profiling

**DOI:** 10.1038/ncomms8440

**Published:** 2015-06-11

**Authors:** Julia M. Rogers, Luis A. Barrera, Deepak Reyon, Jeffry D. Sander, Manolis Kellis, J Keith Joung, Martha L. Bulyk

**Affiliations:** 1 Division of Genetics, Department of Medicine, Brigham and Women’s Hospital and Harvard Medical School, Boston, Massachusetts 02115, USA; 2 Committee on Higher Degrees in Biophysics, Harvard University, Cambridge, Massachusetts 02138, USA; 3 Harvard-MIT Division of Health Sciences and Technology (HST), Harvard Medical School, Boston, Massachusetts 02115, USA; 4 Computer Science and Artificial Intelligence Laboratory, MIT, Cambridge, Massachusetts 02139, USA; 5 Molecular Pathology Unit, Massachusetts General Hospital, Charlestown, Massachusetts 02129, USA; 6 Center for Computational and Integrative Biology, Massachusetts General Hospital, Charlestown, Massachusetts 02129, USA; 7 Center for Cancer Research, Massachusetts General Hospital, Charlestown, Massachusetts 02129, USA; 8 Department of Pathology, Harvard Medical School, Boston, Massachusetts 02115, USA; 9 Department of Pathology, Brigham and Women’s Hospital and Harvard Medical School, Boston, Massachusetts 02115, USA

## Abstract

Transcription activator-like effector (TALE) proteins recognize DNA using a seemingly simple DNA-binding code, which makes them attractive for use in genome engineering technologies that require precise targeting. Although this code is used successfully to design TALEs to target specific sequences, off-target binding has been observed and is difficult to predict. Here we explore TALE–DNA interactions comprehensively by quantitatively assaying the DNA-binding specificities of 21 representative TALEs to ∼5,000–20,000 unique DNA sequences per protein using custom-designed protein-binding microarrays (PBMs). We find that protein context features exert significant influences on binding. Thus, the canonical recognition code does not fully capture the complexity of TALE–DNA binding. We used the PBM data to develop a computational model, Specificity Inference For TAL-Effector Design (SIFTED), to predict the DNA-binding specificity of any TALE. We provide SIFTED as a publicly available web tool that predicts potential genomic off-target sites for improved TALE design.

## Abstract (web summary)

TALE proteins are popular tools for genome engineering because they can recognize specific DNA sequences, however off-target effects are a routine problem. Here Rogers and Barrera *et al*. comprehensively map TALE–DNA interactions to develop a computational model to predict binding specificity.

## Graphical abstract

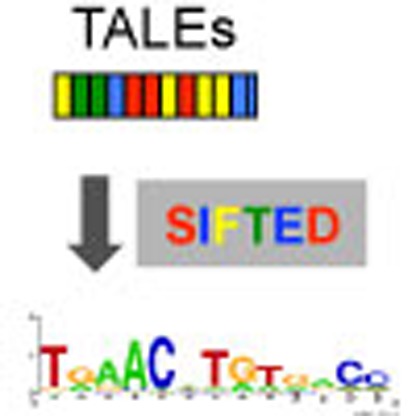

The discovery of Transcription Activator-Like Effector (TALE) proteins has enabled the development of a host of genome and epigenome editing technologies^1^,^2^,^3^,^4^,^5^,^6^,^7^,^8^. Naturally occurring as bacterial virulence factors, TALE proteins harbour an array of repeats, each 33 or 34 amino acids in length^9^,^10^. The sequence of the repeats is highly conserved except at the hypervariable positions 12 and 13, termed the repeat variable diresidues (RVDs). The amino acids at the RVD positions determine which DNA base is preferred, and each repeat in the TALE contacts one base in the target site. This led to a simple one-to-one ‘TALE code’ that uniquely predicts the optimal DNA target from the sequence of RVDs within the repeat array^9^,^10^. The most commonly used RVDs are NI (Asparagine, Isoleucine), HD (Histidine, Aspartic Acid), NN (Asparagine, Asparagine) and NG (Asparagine, Glycine), used to target A, C, G and T, respectively. Co-crystal structures have shown the mechanism of this one-to-one code, in which the TALE protein wraps around the DNA in a helical structure with each repeat contacting a single base^11^,^12^. In addition, contacts between the N-terminal region (NTR) of the TALE protein and DNA specify a preference for a thymine base 5′ to the DNA target site^13^.

This simple TALE recognition code allows for any DNA site preceded by a T to be targeted by a TALE protein designed with the corresponding repeat sequence. Therefore, the TALE DNA-binding domain has been adapted for use in many technologies that require precise targeting of genomic loci. For example, dimeric TALE nucleases (TALENs) have been used in various organisms and cell lines to knock out genes by the introduction of indels or to create specific mutations^2^. Fusions of TALE monomers to transcriptional activation or repression domains can create artificial transcription factors, which have been shown to strongly and cooperatively modulate gene expression^4^,^6^,^8^. Monomeric TALE fusions to chromatin-modifying enzymes can introduce specific DNA or histone modifications at target loci, resulting in changes in expression of the associated genes^3^,^5^. TALEs can also be used to pull down specific genomic regions to identify bound proteins^1^. In addition, TALEs fused to fluorescent proteins can be used to visualize chromatin dynamics in live cells^1^,^7^. Although other technologies, (for example, CRISPR-Cas9) have also been developed for some of these targeting applications^14^, TALE versus dCas9 fusions might be more effective in different applications and having both technologies in the toolkit for genome engineering is likely optimal.

Despite these successes in genome editing, off-target activities of TALE fusions have been described but have proven difficult to predict^15^,^16^,^17^,^18^,^19^,^20^,^21^. Experimental approaches have identified off-target TALEN effects^20^, but no technology has directly measured off-target binding for monomeric TALE fusions^15^,^16^,^17^,^22^. Here, we define TALE protein specificity as the relative binding energies of the protein to different DNA sequences. Computational tools that use the specificities of the individual repeats to predict the specificity of the whole protein have been developed to predict off-target binding sites^23^,^24^; these approaches assume that each repeat independently contributes to the specificity of the whole protein and that each instance of a given repeat RVD type has the same preference for its intended base. However, a quantitative analysis of TALE affinity indicated that repeat position within the repeat array affects RVD specificity, indicating a potential role for repeat context in predicting specificity^25^. Other studies have also found that total protein length affects specificity^20^. In addition, particular repeat types may contribute differentially to overall protein specificity. One study showed that some repeats are more active when assembled into a TALE activator, leading to the distinction between strong (NN and HD) and weak (NI and NG) repeats, although the relationship between RVD strength and specificity is unclear^26^. Altogether, these findings suggest that TALE–DNA-binding specificity may be more complex than previously thought, but these effects have yet to be assayed comprehensively and quantitatively.

Tools used to predict TALE specificity and to identify likely genomic targets have not kept pace with these increasing, albeit qualitative, reports on TALE–DNA recognition. Some computational tools, such as PROGNOS and Talvez, have incorporated context effects qualitatively in predicting TALEN pair off-target sites, but assume all repeat types are affected identically by context^27^,^28^. A recently described approach used a selection-based cleavage assay to characterize a TALEN pair’s specificity profile in order to identify potential TALEN off-target sites; however, that study did not provide a predictive model, but instead required that the specificity of each TALEN pair be determined experimentally^20^. As such, there remains a need for a purely computational tool that quantitatively incorporates these context effects in predicting TALE specificity, and thus, off-target binding sites.

In this study, we perform a quantitative, in-depth examination of context effects on RVD specificity in order to infer general rules for highly accurate prediction of the DNA sequence-specificity of any TALE protein. We design custom protein-binding microarrays (PBMs) to investigate the DNA-binding specificities of 21 TALE proteins that comprise all possible pairs of repeat types. The custom PBMs contain probes in which all possible mono- and di-nucleotide substitutions within the TALE target sites are represented. The resulting quantitative binding data for the TALE proteins to ∼20,000 unique DNA sequences allow us to quantify the effects of TALE repeat array length, repeat position and neighbouring repeat types on the specificity of each RVD, henceforth referred to as RVD specificity. We use the PBM-derived quantitative binding data to develop a computational model (Specificity Inference For TAL-Effector Design or SIFTED) that incorporates these context effects to predict both the DNA-binding specificity and the potential off-target sites of any TALE protein without requiring any additional PBM experiments. We implement this model in a publicly available, user-friendly suite of web tools at http://thebrain.bwh.harvard.edu/sifted.html.

## Results

### Custom-designed PBMs to assay TALE–DNA-binding specificity

**Figure 1:**
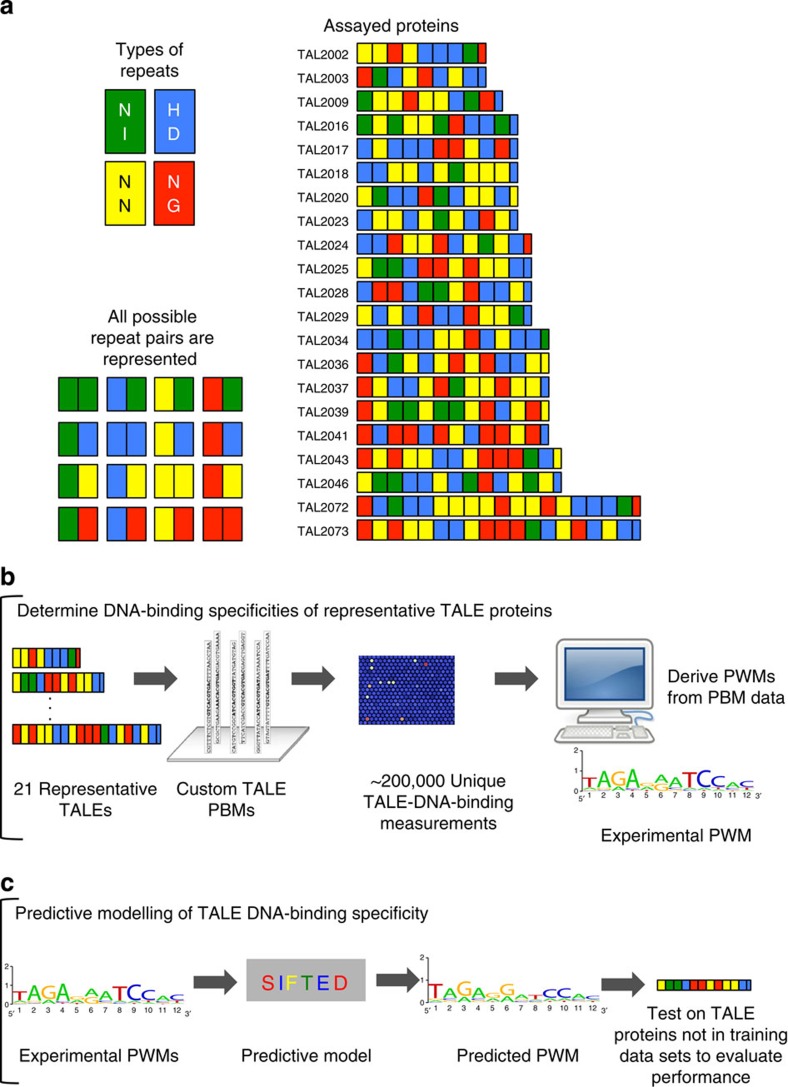
Overall experimental design and analysis scheme. (**a**) 21 Representative TALE proteins used in this study. Repeats are indicated by coloured rectangles, and C-terminal half-repeats are indicated by smaller rectangles. RVD identities are indicated by letters. The set was chosen to include all possible repeat pairs and to cover a range of repeat lengths from 8.5 to 18.5 repeats. (**b**) Custom-designed PBMs were used to determine the specificity of representative TALE proteins. (**c**) These specificity profiles were used to learn features of TALE–DNA recognition and to train a predictive TALE specificity model, SIFTED (Specificity Inference for TAL-Effector Design).

To develop a more in-depth, quantitative understanding of TALE–DNA recognition, we determined the DNA-binding specificities of 21 representative TALE proteins using custom-designed PBMs^29^,^30^,^31^ (Fig. 1a,b, Supplementary Table 1 and Supplementary Data 1). We selected these proteins to allow us to examine the effects of different protein features on specificity. In particular, these proteins represent all possible consecutive repeat pairs and thus allow us to assay all possible direct neighbour effects on RVD specificity (Fig. 1a)^32^. In addition, this set spans protein lengths from 8.5 to 18.5 repeats (targeting sites 10–20 base pairs in length); these lengths typically have been used in the design of monomeric TALE fusion proteins for genomic applications^4^.

PBMs are double-stranded DNA microarrays that permit rapid, highly parallel measurement of the binding of a protein of interest to tens of thousands of unique DNA sequences in replicate, allowing for a much richer picture of TALE–DNA recognition than has resulted from prior studies. As the vast majority of our selected TALE proteins were designed to recognize sequences longer than those on the previously designed ‘all 10-mer’ universal PBM design^30^, we designed custom TALE-PBMs for this study. Each probe sequence was represented on at least eight replicate spots on the arrays. The initial custom array was designed to broadly assay the binding preferences of our representative set of TALE proteins. Subsequently, additional arrays were designed to validate particular observations about TALE specificity, as discussed below (Supplementary Fig. 1 and Supplementary Note 1).

We determined the DNA-binding specificities of each TALE protein using probe sets that contain each protein’s target site as predicted by the canonical TALE code^9^, as well as variants thereof, flanked by constant DNA sequence and situated at a fixed position within the probe relative to the slide surface (Supplementary Note 1). The constant flanking sequence was predicted to not be bound by any of the TALEs tested in this study. For each protein, we measured binding to between 160 and 320 variant target sites that cover all possible adjacent dinucleotide substitutions. Although the absolute *K*_d_ of a protein–DNA interaction cannot be determined from a single PBM experiment^33^, by measuring how much each substitution changes protein binding to the DNA probe, we can infer changes in binding free energy (ΔΔ*G* values) for each possible substitution within the target site.

**Figure 2:**
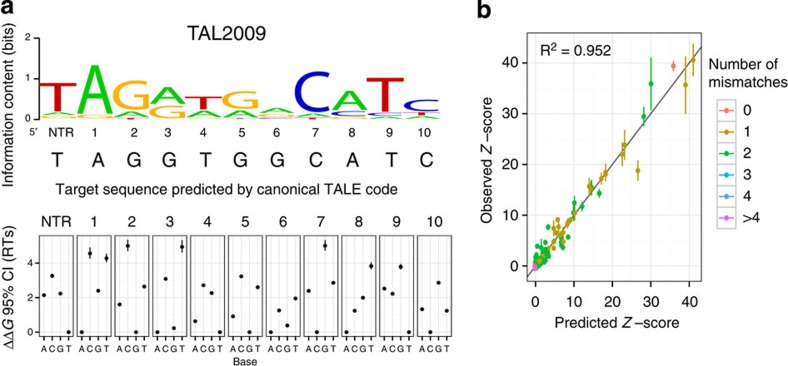
Determining PWMs from custom-designed PBMs. (**a**) Representative logo and ΔΔ*G* estimates. The vertical bars represent the 95% credible interval (CI) and the points show the mean of the posterior distribution, in units of RT. The base predicted for each position by the TALE code is indicated below the logo. (**b**) Representative comparison between the probe *z*-scores measured in PBMs and the *z*-scores predicted by the derived PWM. Points represent the mean and vertical bars show its 95% CI. Points are coloured by the number of mismatches between the sequence in the probe and the consensus sequence predicted from RVD identities using the canonical TALE code. NTR, N-terminal region.

From these ΔΔ*G* values, we derived a position weight matrix (PWM) for the protein (Fig. 2a and Supplementary Fig. 2). The inferred PWMs were consistent across experimental replicates and across PBM experiments performed at different concentrations of TALE proteins (Supplementary Fig. 3). Our PWMs accurately predict the 60-base-pair probe signal intensities, with a median *R*^2^ of 0.959 (Fig. 2b and Supplementary Fig. 4), indicating that they perform well as accurate models of TALE–DNA-binding specificity.

The fact that our PWMs explain binding well suggests that an additive binding model with independence between the nucleotides in the TALE target site is quite accurate. To test if this nucleotide independence extends beyond two adjacent mismatches, we designed a probe set that contains up to five nonadjacent mismatches in the target site (Supplementary Note 1 and Supplementary Fig 1). The PWM models derived from the dinucleotide substitution probes accurately predicted binding to these sequences with additional mismatches (median *R*^2^ greater than 0.9 for all numbers of substitutions tested), indicating that the simple PWM models with mononucleotide independence perform well in modelling TALE–DNA-binding specificity (Supplementary Fig. 5). These results are roughly consistent with a recent study of TALEN pair specificity determined by a selection-based cleavage assay, in which general independence in DNA recognition was observed; however, our data support a fully independent model of TALE–DNA binding, rather than a model with slightly increased tolerance for adjacent mismatches^20^.

### Modelling repeat context improves specificity prediction

Although we observed mononucleotide independence within TALE target sites, we found that the protein–DNA interactions of a given repeat are influenced by its context. In other words, the energetic parameters of a given TALE–DNA contact are not affected by neighbouring nucleotide changes, but they are affected by the repeat context. Intriguingly, even within a single TALE protein, different occurrences of the same repeat type can exhibit very different specificities. For example, in TAL2009, repeats 7 and 10 were both designed with the HD RVD to target C, but within the context of the TAL2009 protein each exhibits substantially different relative preferences for C as compared with other nucleotides (Fig. 2a). Typically, the highest scoring probe corresponded to the target sequence predicted by the canonical TALE code; however, we observed multiple cases (for example, TAL2024) where a TALE protein bound mismatched sequences with comparable binding strength, hereafter referred to as affinity. Moreover, some TALEs (for example, TAL2009) even preferred a mismatched sequence to the predicted optimal target sequence; this most frequently involved an NN RVD, which can target both a G and an A in different contexts (for example, see repeats 3 and 6 in Fig. 2a)^10^. Altogether, these results highlight that the simple one-to-one TALE code is not sufficient to accurately predict DNA-binding specificity.

As our results suggested that interactions between repeats modulate their individual RVD specificities, we modelled the PBM data to predict TALE specificity considering the context of each repeat in a TALE protein (Fig. 1c). We named our model and its associated software tools SIFTED (Specificity Inference For TAL-Effector Design). In addition to modelling the intrinsic specificity of each RVD, SIFTED considers a variety of repeat context features, including the number of repeats in the protein, each repeat’s position within the repeat array, and the immediately adjacent N- and C-terminal neighbouring repeat types. The NTR, which specifies the preference for the 5′ T in the binding site, was also included in the model and was treated equivalently to a repeat, except for the omission of its position and length features.

We trained the SIFTED model by performing a linear regression with Elastic Net regularization, using the ΔΔG values inferred for each protein as the input data^34^. To prevent overfitting and to assess performance, we used a nested leave-one-out cross-validation strategy. Briefly, one protein was held out from the data set in an iterative manner. The remaining proteins were divided into training and test sets, which were used to derive parameter values and to control the complexity of the model (Supplementary Fig. 6). The predicted PWM for each of the 21 TALE proteins was obtained from the model trained on data from the remaining 20 proteins in our data set (Fig. 1a). For specificity predictions of proteins not in our data set (for example, TALEN pairs), the regression was performed on the full data set (no proteins excluded) and the resulting model was used to make PWM predictions.

**Figure 3:**
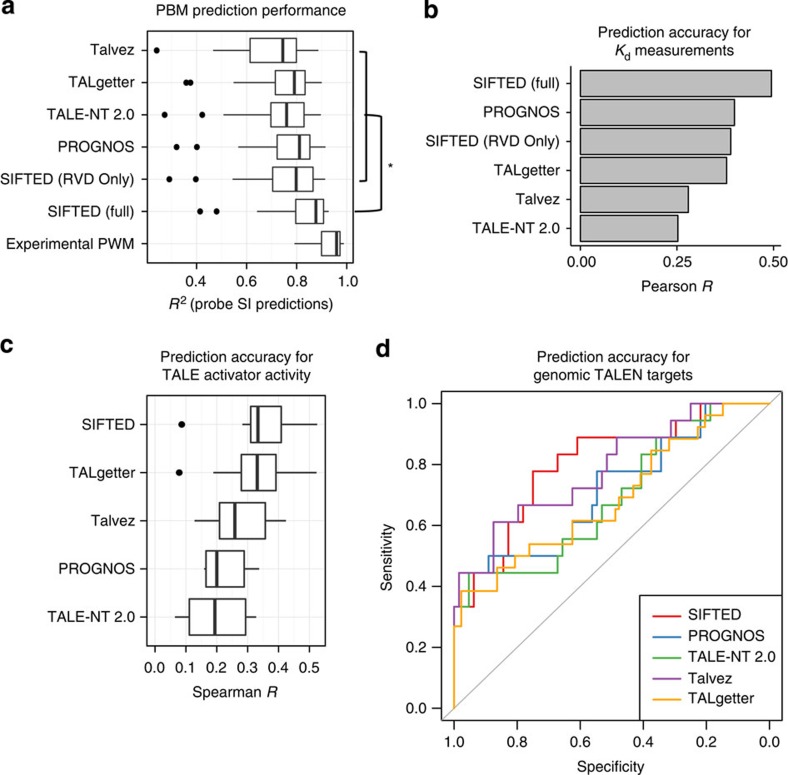
SIFTED predictive model performance. (**a**) Comparison of prediction accuracy of PWMs derived by different methods. The box plots show how well the PBM probe intensities for each protein are predicted by the PWMs generated by SIFTED and other methods. Two versions of SIFTED are shown: one that only models repeats independently (‘SIFTED (RVD Only)’) and one that considers all repeat context features (‘SIFTED (Full)’). Experimental PWMs are those derived from the PBM data. (*) The brackets highlight a subset of statistically significant differences between the full SIFTED model and each of the models shown inside the top bracket (*P*<10^−6^, Wilcoxon signed-rank test). The box plots shows the median and the first and third quartiles. Whiskers extend to data points not considered outliers, whereas outliers are shown as individual points. Data are considered outliers when they are 1.5 times the interquartile range (IQR) higher than the third quartile, or 1.5 * IQR lower than the first quartile. (**b**) Prediction accuracy for relative binding affinity. PWMs derived from existing tools or from SIFTED (as in **a**) were used to predict relative *K*_d_ values for a single TALE protein^27^,^35^. The bars display the Pearson correlation coefficient between observed and predicted log(*K*_d_) values. (**c**) Validation of TALE activator binding specificity predictions by comparison to TALE activator activity data reported in Mali *et al*^22^. The five predictive methods were used to score all reported binding sites up to three mismatches away from the predicted target. These scores were compared with an expression score associated with that binding site using Spearman correlation. (**d**) Validation of TALEN-binding specificity predictions by comparison to cell-based TALEN activity data, reported in Guilinger *et al*^20^. The five methods shown were used to predict the binding of TALEN pairs to genomic target sites. The receiver operating characteristic curves show the sensitivity and specificity of each method for distinguishing genomic sites that showed nuclease activity (that is, indels) and those that did not.

**Figure 4:**
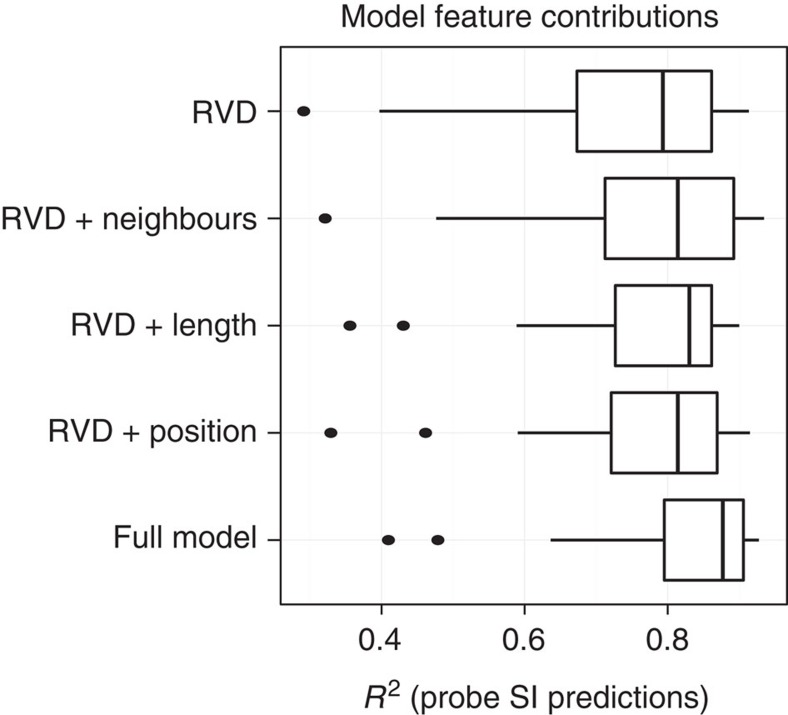
Contribution of model features. The plot shows the accuracy at predicting PBM probe intensities of a PWM predicted with no context features (top), with one single context feature added (middle) or with all context features included (bottom). Box plots are formatted as in Fig. 3a.

To assess how well our model explains binding, we used the PWMs obtained from the cross-validated SIFTED model to predict PBM probe signal intensities. The SIFTED PWMs accurately predict the probe-level PBM-binding data (median *R*^2^=0.877). In addition, SIFTED outperformed the specificity models from other available computational tools designed to predict off-target sites in explaining the PBM data (*P*<10^−6^, Wilcoxon signed-rank test; Fig. 3a). Two of these tools, TALE-NT 2.0 (ref. 23) and TALgetter^24^, do not consider any context effects. Others, such as PROGNOS^27^ and Talvez^28^, include context effects on an RVD’s specificity only as discrete penalties. In contrast, SIFTED models context effects quantitatively and also allows each repeat type (that is, NI, HD, NN and NG) to be influenced differently by its context. These detailed context parameters in our model are keys to its success; the full model predictions from SIFTED are more accurate (*P*<10^−6^, Wilcoxon signed-rank test) than those of an RVD-only model that represents the canonical, one-to-one TALE–DNA recognition code (median *R*^2^=0.798; Fig. 4).

We validated that our SIFTED model can predict off-array binding affinity measurements (*K*_d_ values) more accurately than other published tools^35^ (Fig. 3b). Although PWMs cannot be used to predict absolute dissociation constants, they are able to predict the affinity of a sequence relative to that of the optimal binding site (that is, relative *K*_d_ values)^36^. The full SIFTED model performed significantly better than PROGNOS, TALE-NT 2.0, TALgetter, Talvez or a reduced SIFTED model with no context effects in predicting relative *K*_d_ values for 1 protein and 18 DNA sequences^35^.

### Quantitative modelling of context effects on RVD specificity

**Figure 5:**
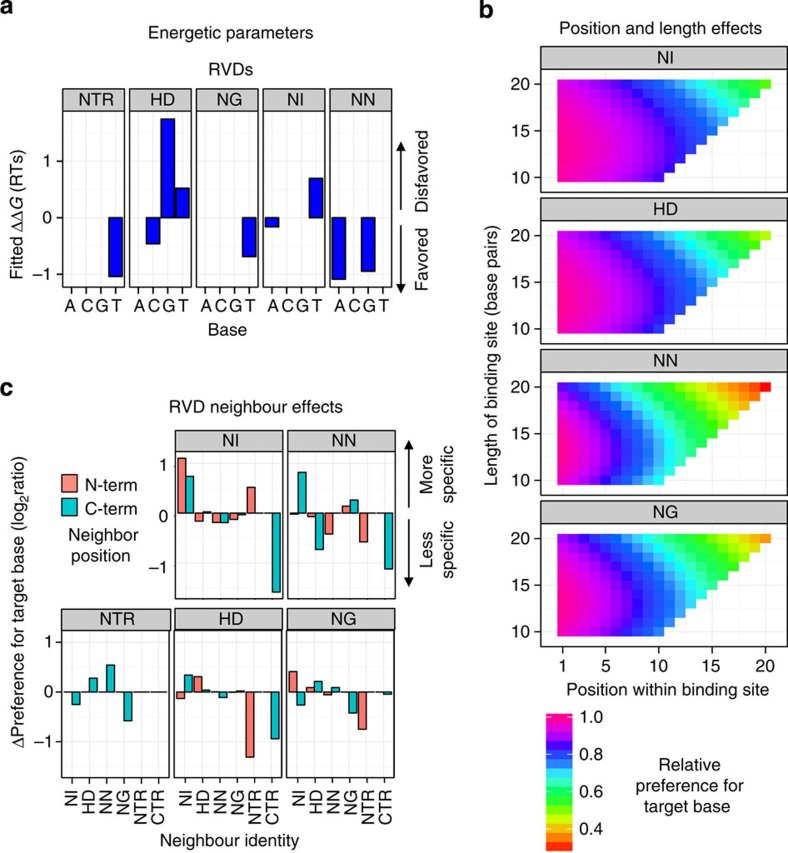
Protein features that affect repeat specificity. (**a**) RVD identity. ΔΔ*G* values from the model are indicated for each repeat type with each base. In addition, the ΔΔ*G*s for the four bases at the 5′ T position, which are contacted by the N-terminal region (NTR), are shown. (**b**) Position and length. The effects of repeat position and protein length on the specificity of each repeat type are shown. (**c**) Effect of neighbouring repeats or terminal regions on specificity. For each repeat type and the NTR, the bar heights display the effect on specificity for different neighbours in the N- or C-terminal direction (orange and teal, respectively). The quantity shown is the log_2_ ratio between the PWM frequency predicted with and without the presence of a given neighbour in the model. CTR refers to the C-terminal region; repeats with the CTR as the C-terminal neighbour are the half-repeats in the final repeat position.

As context effects contributed significantly to the predictive power of our model, we investigated in greater depth how length, position and neighbouring repeats each affect specificity. Although our baseline RVD specificities (Fig. 5a) largely agree with previous studies^9^ (for example, NN is the least specific RVD and can target both G and A), in the SIFTED model these specificities are modulated by the protein context of each instance of the repeat.

Our data are consistent with previous reports that longer proteins tolerate more mismatches in their target sites^20^ (Fig. 5b). Our comprehensive profiling also revealed that NN and NG repeats are affected more strongly by protein length than are either NI or HD. In addition, our set of proteins included two proteins of different lengths designed to target overlapping sites. The longer protein (TAL2073) is less specific overall (that is, lower total information content) than the shorter protein (TAL2043; Supplementary Fig. 2), directly supporting our overall finding that increased TALE protein length diminishes RVD specificity.

Repeat position within the repeat array also affects the specificity of C-terminal repeats that target the 3′ end of the DNA binding site, resulting in their being more tolerant to substitutions than N-terminal RVDs. To test this modelling result, we designed a custom PBM that included probes containing clusters of three nucleotide substitutions located at either the 5′ or 3′ end of the target site (Supplementary Fig. 1 and Supplementary Note 1). In general, substitutions at the 5′ end impaired binding more than substitutions at the 3′ end (*P*<0.05, Wilcoxon signed-rank test; Supplementary Fig. 7), supporting prior observations from reporter assays^25^,^37^. Talvez and PROGNOS model this polarity effect discretely as a constant decrease in specificity after a certain position in the repeat array for all repeat types^27^,^28^. In contrast, SIFTED continuously models the decrease in specificity over the length of the protein and allows different repeat types to be affected to different extents (Fig. 5b).

Last, we observed that a repeat’s specificity is impacted by the identity of the immediately adjacent N- or C- terminal repeat (Fig. 5c). Such local context effects previously have been observed only for the 5′ T preference, which is more important for binding when the first repeat is an HD^38^. We also observed the influence of HD in the first position, but found an even stronger effect when the first repeat is an NN. In addition, we observed neighbour context effects between repeats within the protein. For example, the NN repeat is more specific for targeting a G when the NI repeat is either N- or C-terminal to it; however, it is much less specific for G when it is positioned at the C-terminal end of a TALE repeat array.

We found that a particular repeat type can exert different effects as an N- or C-terminal neighbour (Fig. 5c). PROGNOS includes a parameter to reduce an RVD’s specificity when it is next to a strong RVD (NN or HD), positing that a stronger neighbouring interaction may allow for greater mismatch tolerance^26^,^27^; however, it does not distinguish between N- and C-terminal neighbours. The neighbour effects we found are more complex, and in fact, the strong RVDs do not always decrease specificity. The complexities of the neighbouring effects are captured quantitatively in SIFTED; each of the four RVDs as well as the 5′ T preference are modelled as being affected differently by its N- and C-terminal neighbouring repeats.

These observations of context effects can be condensed into some simple guidelines for TALE design (Table 1). Certain repeat combinations (for example, NI–NI) are predicted to have increased specificity, whereas others (for example, NG as the N-terminal repeat) can make an RVD more tolerant to mismatch and therefore should be avoided. However, when designing TALE proteins, one must ultimately consider all the context effects in the protein, as well as the prevalence of potential off-target sites in the genome. As such, we tested if the SIFTED model could accurately predict genomic off-target sites, and therefore could be used to guide TALE protein design.

### Predicting TALE off-target sites using SIFTED

To assess whether SIFTED can predict genomic off-target sites for TALE proteins that have not been assayed by PBMs, we examined a data set of *in vivo* TALE reporter activity^22^. SIFTED had the highest median performance of the five tools tested (Fig. 3c).

Although SIFTED was designed to predict TALE monomer specificity, we also tested its ability to predict TALEN binding by examining a large data set of TALEN activity in cells^20^. We derived the specificities of TALEN pairs from the specificities of the component monomers predicted by SIFTED. The PWMs from SIFTED resulted in better sensitivity and specificity than those from any of the other models in distinguishing genomic target sites that showed nuclease activity from those that did not (Fig. 3d and Supplementary Data 2). The area under the receiver operating characteristic curve statistic was used to quantify the ability of the five tools to distinguish target from non-target sites across all possible score thresholds. SIFTED demonstrated superior sensitivity and specificity across most thresholds.

In addition, we considered that a typical TALE user might investigate about 20 off-target sites when analysing the specificity of their designed protein in their genome of interest. To provide a performance comparison for this typical use case, we investigated how many of the top 20 off-target sites predicted by these tools have been identified as TALEN pair off-targets *in vivo* (Supplementary Fig. 8a,b) or were among the 20 off-targets with the highest measured *in vivo* activity (Supplementary Fig 8c). Again, SIFTED performed better than the other tools, demonstrating higher sensitivity by predicting more of the true off-targets than the other tools (Supplementary Fig. 8b and Supplementary Data 2).

### Prediction of genomic off-targets with SIFTED web tool

SIFTED was the top-performing model overall, highlighting the value of incorporating repeat context effects in predicting specificity. Although other tools may perform comparably to SIFTED in a specific application, SIFTED was the only tool that was consistently a top performer across the wide range of benchmarks of predictive performance (Fig. 3 and Supplementary Fig. 8). Given the success of SIFTED in predicting off-target binding, we developed it into a web-based suite of tools to aid in TALE design implemented on the Galaxy platform^39^,^40^,^41^ at http://thebrain.bwh.harvard.edu/sifted.html. We provide stand-alone tools for individual tasks, such as predicting the specificity and genomic binding sites of a user-specified TALE, as well as a pipeline that combines various tools to automate the process of designing a TALE to target a particular genomic region. The complete pipeline takes a user-defined genomic target region as input, and then (i) identifies candidate TALEs to target that input region, (ii) predicts the candidates’ specificities, (iii) finds instances of off-target sites in a user-specified genome and (iv) outputs a list of candidate TALE proteins ranked by their off-target binding potential, thus allowing the user to select the best candidate protein.

## Discussion

By analysing TALE proteins of different lengths and containing all possible consecutive pairs of repeats, we were able to identify the influence of repeat context on DNA-binding specificity. In contrast to other studies that used cell-based TALEN activity as a measurement of TALE specificity^35^, our experimental design allowed us to directly assay the intrinsic binding properties of TALE monomers. We measured a total of ∼200,000 binding interactions between 21 TALE proteins and ∼5,000–20,000 unique DNA sequences per protein using custom-designed PBMs. Importantly, the resulting data set allowed us to develop a model to predict TALE specificity for any candidate TALE protein without requiring any additional experimental analysis.

Our results highlight that RVD specificity is not determined simply by what base a particular RVD will bind, but also which bases it strongly disfavours. This information could be useful in designing TALEs for allele-specific applications, such as rapid, spatially resolved genotyping of patient samples through binding of fluorescently tagged, allele-specific TALEs. The HD RVD has the greatest power to discriminate between two alleles: it prefers binding to a C and strongly disfavours binding to a G. Therefore, targeting an allele where there is a C/G SNP may lead to stronger discrimination between the two alleles.

We found that longer TALEs are generally less specific than shorter TALEs. This effect could be due to excess DNA-binding energy in TALE proteins with many repeats^20^. The mechanism of the context effects on RVD specificity remains to be determined. An ability to tolerate some binding site mismatches may allow a TALE protein from xanthomonad pathogens to overcome mutations in host genomic target sites, as the plant host may be under selection to escape xanthomonad infection. However, TALEs with very low specificity may lead to potential negative effects on virulence because of additional binding in the host genome^42^. Thus, the specificity of TALE proteins may have been strongly shaped by the complex interactions between host and pathogen.

SIFTED predicts that some DNA sequences should be targeted with greater specificity, which could be interpreted as guidelines for TALE design (Table 1). Interestingly, some of these guidelines would contradict published guidelines that were developed as part of the SAPTA tool for designing more active TALEN pairs^43^. For example, although we predict that A-runs can be targeted with high specificity by TALE monomers, SAPTA predicts that TALENs targeting A-runs will have lower nuclease activity. The discrepancies in these guidelines and results might reflect different rules affecting the binding of monomeric TALEs versus dimeric TALENs. Alternatively, it is possible that a trade-off exists between optimizing activity and specificity in designing TALENs. Previous reports have found no correlation between activity and affinity^35^. This lack of correlation between *in vitro* binding and different cell-based activity measurements might be due to other genomic features in cells, such as the chromatin state and competition with other transcription factors at the target and off-target sites. Ultimately, in designing TALEs, the intrinsic specificity of the protein must be considered in light of its potential off-target binding sequences in the genome. For example, the decreasing specificity of longer TALEs may be compensated by longer target sites being more rare in the genome, thus increasing the effective specificity of a protein^20^. SIFTED can both model protein specificity as well as identify genomic off-target sites, revealing the effective specificity of a TALE, so users can choose the most specific TALE protein for their particular application.

Future studies will be required to identify chromatin features that might modulate binding specificity *in vivo*. In addition, the specificities of other alternative RVDs (for example, NH to target G) could be studied to enable design of TALE proteins with higher sequence specificity. An improved understanding of TALE–DNA binding should allow for development of more precise genome engineering tools.

## Methods

### Cloning of TALE proteins

TALEN expression vectors^32^ were digested with *Sac*II and *Bam*HI to obtain the DNA-binding domain comprising the Δ152 N-terminal domain, the RVD repeats, and the +63 C-terminal domain. This fragment was ligated into a modified pDONR221 vector (Invitrogen), with *Sac*II and *Bam*HI restriction sites internal to attL recombination sites, to create Gateway-compatible TALE Entry clones. The TALE constructs were then transferred by Gateway recombinational cloning into the pDEST15 expression vector, which adds an N-terminal glutathione S-transferase (GST) tag (Invitrogen), by an LR reaction. All clones were full-length sequence-verified (Supplementary Data 1).

### Custom PBM design

Target sites for each TALE protein were determined using the canonical TALE code (NI: A, HD: C, NN: G, NG: T), and are preceded by the 5′ T to create the full target site. The constant flanking regions were the same as that used in a prior custom PBM design and do not contain binding sites for any of the TALE proteins in this study^44^. Probe set descriptions, including the array design versions on which they are included, are provided in Supplementary Note 1.

### PBM experiments

Proteins were expressed using the PURExpress *In Vitro* Transcription and Translation Kit (New England Biolabs). Protein concentrations were determined by anti-GST western blots with a dilution series of recombinant GST (Sigma). Proteins were stored at +4 °C until being used in PBM assays. PBMs were performed as follows:^29^ briefly, custom-designed microarrays were first double-stranded by an on-slide primer extension reaction. In the PBM assay, arrays were blocked with 2% milk in PBS for 1 h, washed with 0.1% Tween-20 in PBS and 0.01% TX-100 in PBS, then incubated with protein mixture (PBS, 2% milk, 0.2 mg ml^−1^ BSA and 0.3 μg ml^−1^ salmon testes DNA) for 1 h. The final concentration of TALE protein in the PBM reactions was 200 nM, unless otherwise indicated (Supplementary Table 1). Arrays were washed with 0.5% Tween-20 in PBS and 0.01% TX-100 in PBS. Lastly, the array was incubated for 30 min with an Alexa488-conjugated anti-GST antibody (Invitrogen A-11131), and washed with 0.05% Tween in PBS and PBS.

### PBM data quantification

PBM arrays were scanned using a GenePix 4400A Microarray Scanner (Molecular Devices), and scan images were analysed by GenePix Pro (Molecular Devices). Raw data files were processed using the same general approach as used for universal PBMs^29^. Briefly, masliner software^45^ was used to combine Alexa488 scans at three different laser power levels and to resolve the signal intensity in spots that are saturated at high laser power settings. Cy3 scans were performed at a single laser power level. If a data set had any negative background-subtracted intensity (BSI) values (which can occur if the region surrounding a spot is brighter than the spot itself), a pseudocount was added to all BSI values for that experiment such that all values were then positive. The adjusted BSI data were then normalized by the corresponding double-stranded DNA content of the spots and their position on the array using the same approach as described for universal PBMs^29^. To normalize by the relative amount of double-stranded DNA per array spot, small quantities of Cy3-dUTP were added to the nucleotide pool during the double-stranding process. The BSIs on the Cy3 channel can therefore be used to estimate relative DNA abundance at each spot. However, because Cy3 incorporation depends on the local sequence context, we used a linear regression over the trinucleotides present in a given probe to calculate the expected Cy3 BSI and obtain the expected-to-observed ratio^30^. This ratio is then used to normalize the Alexa488 BSIs to account for difference in relative amounts of double-stranded DNA. Any probes with BSIs that were corrected by more than twofold or for which the adjustment would lead to a negative BSI value were removed from the data.

All PBM designs include at least eight replicate probes for each sequence. For each experiment and for each set of probes with identical sequences, we calculated the median-adjusted BSI, median absolute deviation (MAD) and the robust standard deviation estimate from the MAD. Any individual replicate probe with a normalized adjusted BSI value more than 3 s.d. away from the median of the replicate probes was omitted from subsequent analysis, to avoid confounding statistical tests or incorrect choice of parameter settings in model fitting.

For each TALE protein, we defined a background set of probes that comprises all the probes on the array designed to represent binding sites for other TALE proteins (not the one being assayed in a given experiment). The array median level was then calculated as the median normalized adjusted BSI of all probes in the background set. The standard deviation of the background set SIs was calculated robustly using the asymptotic approximation *σ*=1.4826 × MAD. The *z*-score for each probe was calculated relative to the median and standard deviation of its corresponding background probes. These *z*-scores represent a linear transformation of the median SIs for each probe, and therefore facilitate interpretation but do not affect the PWM fitting procedure, which performs its own linear scaling adjustments.

### PWM model fitting

We developed a Bayesian Markov chain Monte Carlo (MCMC) method to infer free energy parameters of TALE–DNA interactions from PBM data. We relied on the theoretical framework developed for the BEEML-PBM algorithm^36^, which can accurately derive ΔΔ*G* values for protein–DNA contacts from universal PBM experiments. The BEEML-PBM framework estimates ΔΔ*G* values for each possible nucleotide substitution in a protein’s DNA-binding site motif. These values can be assembled to construct an energy matrix (EM), in which each column represents a position within the binding site and each row represents a nucleotide. The EM values can be converted to probabilities using the Boltzmann distribution, creating a PWM.

Briefly, the goal is to predict the observed probe signal intensity *z*-scores as a function of the binding site sequence within the probe. As an intermediate step, the ΔΔ*G* values are used to predict occupancy of the TALE protein on its binding site. The predicted occupancy is then scaled linearly to optimally scale with the observed *z*-scores. The chemical potential (log([TF]/*K*_d_) was also included in the model and can account for differences in TALE protein concentration and affinity to the optimal binding site. The statistical model is described in full in Supplementary Methods. At each sampling step, the probe *z*-scores are predicted given the current parameter values, which can be used to derive 95% credible intervals^46^, as shown in Fig. 2a. The priors on ΔΔ*G* values were set as exponential distributions with mean 10.0 to cause the preferred base to adopt values close to 0 but to not significantly penalize larger parameter values for other bases. The rest of the parameters were given a uniform prior.

To perform MCMC sampling, we used the No-U-Turn Sampler included in Rstan v2.0 with default parameter settings. The ΔΔ*G* parameters were initialized following a simple TALE code: ΔΔ*G*=0.0 for the predicted optimal base at a given position, ΔΔ*G*=3.0 otherwise (in units of kT/RT). For each data set, we obtained 500 parameter samples in the burn-in period followed by 2,000 samples that were used to approximate the posterior distributions of all parameters. Four MCMC chains were run in parallel for each data set; the samples from each chain were then used to verify convergence of all ΔΔ*G* parameters (Gelman-Rubin convergence statistic for all four chains <1.05). Note that sampling is more efficient in Hamiltonian MCMC methods (such as No-U-Turn Sampler) and thus fewer iterations are required than in standard MCMC methods, such as Gibbs sampling^47^.

### SIFTED predictive model for ΔΔ*G* values

The ΔΔ*G* values inferred from the TALE PBM experiments were used to train a predictive model using an Elastic Net regression^34^. The energy term for each inferred protein–DNA contact (that is, each repeat contacting each of the four possible nucleotides) represents a single observation. However, each column in the EM has only three degrees of freedom, since adding a constant value to all terms does not change the resulting PWM. Therefore, each EM derived from the data was first adjusted by adding a constant value to each column such that the preferred base has a ΔΔ*G* of exactly 0 (because of the exponential prior described above, these values are already close to 0 when the repeat binds its expected base preferentially). These zero-valued ΔΔ*G* terms were then removed from the data set, leaving only the values for the non-preferred bases as input.

The full predictive feature matrix was normalized such that each column had mean zero and unit variance. Numerical features (for example, total length of the target site) are included directly in the feature matrix. In the case of categorical features (for example, RVD identity), we created binary indicator variable columns (‘dummy variables’) representing each potential categorical value. We used regression weights to reduce the biases that could be created by having an unequal number of proteins of different lengths. Each squared error term in the Elastic Net objective function was multiplied by the weight corresponding to that observation. The observations corresponding to a given protein were assigned a weight of 1/(# of proteins of the same length).

We used the Elastic Net implementation in the glmnet v1.9-5 R package to train our model. The Elastic Net is a regularized regression method that seeks to penalize models that are too complex (that is, have too many parameters) and thus prevent overfitting^34^. The Elastic Net objective combines the penalty terms used in L_1_ (or LASSO) and L_2_ (or Ridge) regressions. Here, we set the balance between the two terms to 95% L_1_ penalty and 5% L_2_ penalty, favouring the sparseness of the L_1_ method but also keeping some of the advantages of the L_2_ method, such as the uniqueness of solutions.

Each ΔΔ*G* in the data set is paired with a vector of predictive features to create the feature matrix, in which each row is an independent observation, and each column is a different feature. The features include repeat identity, position, neighbouring repeat identity and total length of the target site. Numerical features (for example, total length of the target site) are included directly in the feature matrix. In the case of categorical features (for example, RVD identity), we created binary indicator variable columns (‘dummy variables’) representing each potential categorical value. To allow for non-linear position and length effects, we also included the natural logarithm of each as a feature. The full predictive feature matrix was normalized such that each column had mean zero and unit variance. We used regression weights to reduce the biases that could be created by having an unequal number of proteins of different lengths. Each squared error term in the Elastic Net objective function was multiplied by the weight corresponding to that observation. The observations corresponding to a given protein were assigned a weight of 1/(# of proteins of the same length).

To prevent overfitting and to accurately assess the model’s performance, we used a cross-validation scheme consisting of two nested levels. On the outer level, we used leave-one-out cross-validation to form a validation set by excluding a single protein in each iteration. Once a protein is excluded, the inner level performs fivefold cross-validation on the remaining 20 proteins. For 100 different values of the Elastic Net penalty term *λ*, we calculate the mean-squared error (MSE) on the test set for the model obtained from the training set (Supplementary Fig. 6). For a given value of *λ*, the average MSE over all test sets is calculated. The *λ* value that minimizes the overall average MSE is then used for all subsequent predictions. This is achieved by creating bootstrap estimates of the MSE at each value of lambda and picking the simplest (that is, most penalized) model that performs within one standard deviation of the model with the lowest average MSE (dashed vertical lines in Supplementary Fig. 6). The model associated with the best *λ* value was then used to make predictions on the protein excluded in the outer loop; the same *λ* value was used for all training sets. This entire process is repeated for each protein, leading to cross-validated predictions for the entire data set. These predictions were then used for all model evaluation purposes.

### Predicting probe signal intensities and *K*_d_ values from PWMs

The predictions of probe signal intensities were obtained using the same mathematical framework as for fitting PWMs (Supplementary Methods). However, in this case, the ΔΔ*G* parameters are known and the only parameters that need to be fitted to predict probe intensities are the chemical potential *μ* and the scaling terms *a* and *b.* To determine these parameters, we used the implementation of the Levenberg–Marquardt algorithm in the SciPy v0.12 package with default convergence parameters. The model parameters were initialized as follows: *a*=minimum z-score in input data, *b*=maximum *z*-score in input data, *μ*=−1.0. After these parameters were fitted from the observed *z*-scores, the predicted *z*-scores were obtained by using the total ΔΔ*G* for the binding site in each probe and the fitted variables as input.

To validate SIFTED predictions with measured *K*_d_ values^35^, relative *K*_d_ values for target and off-target sites were predicted from SIFTED PWMs. Relative *K*_d_ values were predicted by setting the *K*_d_ of the optimal site to 1. The predicted *K*_d_ values for off-target sequences were obtained through the equation e^ΔΔG/RT^, where ΔΔ*G* represents the difference in total free energy between the optimal binding site sequence and the sequence of the off-target site. The measured relative *K*_d_ values were similarly adjusted so that the optimal site had a *K*_d_ of 1. Because *K*_d_ values span many orders of magnitude, the correlation coefficient was computed after taking the natural logarithm of the *K*_d_ values, which prevents the calculation from being dominated by the extreme values.

### Comparison using PWMs from other tools

PROGNOS, TALgetter, Talvez and TALE-NT 2.0, the publicly available tools against which we compared SIFTED, do not explicitly provide the user with predicted PWMs^23^,^24^,^27^,^28^. However, with the exception of TALgetter, each tool uses an internal scoring scheme that is mathematically equivalent to a PWM (that is, the score for a site represents the sum of an independent score for each nucleotide position). Therefore, in the comparisons with PROGNOS, Talvez and TALE-NT 2.0, we predicted PWMs based on the scheme described by each paper and the associated parameters^23^,^27^,^28^. To predict TALgetter scores, we instead used the downloadable TALgetter software tool to compute log-odds values for all binding site sequences in a given experiment^24^. These binding scores can then be compared directly to PWM log-odds scores, even if the underlying scoring scheme is distinct. For comparisons using TALEN activity data, we combined the values predicted by PWMs for each TALE in a TALEN pair using the same scoring scheme as PROGNOS^27^. Here, the scores *S* are obtained by taking the negative natural logarithm of each value in the PWM, creating a value that becomes larger the more disfavoured a particular nucleotide is at a particular position. Then, we compute the ratio of the score *S* summed over the optimal target site and the score *S* summed over the potential off-target site being analysed. These ratios are elevated to an exponent (0.6, as determined to be optimal by Fine *et al*.^27^). Finally, the partial score for each member of the TALE pair is added to create a final score, as in the equation below.




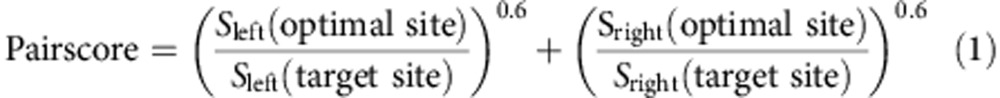




We analysed the TALEN target sites reported by Guillinger *et al*.^20^. We scored each reported target site that contained only NN, NI, HD, and NG RVDs using the TALEN Pair Score derived from the PWMs obtained from SIFTED, PROGNOS and TALE-NT 2.0. We summarized the performance of each tool as a receiver operating characteristic curve, which shows the sensitivity and specificity values achieved by each tool when predicting sites that were targeted by the TALEN pairs. The different sensitivity and specificity values represent different Pair Score thresholds, above which a locus is predicted to show evidence of nuclease activity (indels).

We also compared against the TALE activator reported by Mali *et al*^22^. All of the reported binding sites up to three mismatches away from the predicted site were scored as described above. These scores were then compared with a normalized expression score (the ratio of barcode tags for that binding site relative to a control experiment) associated with that binding site—TALE combination. As we expect the relationship between TALE occupancy and expression to be nonlinear, we compared the results using Spearman correlation.

### Algorithmic approach of SIFTED web tool

The overall approach of the entire pipeline to identify and score candidate TALEs to target a genomic region is as follows: first, candidate TALE-binding sites within the user-input DNA sequence are identified. For each site found, the protein that targets that sequence is determined using the TALE code, and its PWM is predicted. For each protein, the PWM is used to enumerate all putative binding site sequences (both target and off-target sequences) with a relative *K*_d_ threshold (by default, set to 10), using a bounded breadth-first search. All genomic instances of the putative binding site sequences are found using a short read aligner (*bowtie*). Finally, a summary score is calculated for each protein that describes the overall number and strength of genomic target sequences. Under default parameter settings (for example, 13.5 repeat TALE, 1-kb region), the SIFTED pipeline typically identifies optimal TALE candidates within minutes. In addition, a user can input a TALE with a defined RVD sequence, and SIFTED will predict its specificity and identify potential genomic off-target sites. Tutorials are hosted on the SIFTED website (http://thebrain.bwh.harvard.edu/sifted.html) for designing TALEs to target a region, and for predicting the specificity of a pre-designed TALE, and include additional guidelines for setting parameters and troubleshooting. For more details on the algorithmic approach, see Supplementary Methods.

## Additional information

**Accession codes:** All analysed microarray data and array designs have been deposited in NCBI GEO under Series ID 56978.

**How to cite this article**: Rogers, J. M. *et al*. Context influences on TALE-DNA binding revealed by quantitative profiling. *Nat. Commun.* 6:7440 doi: 10.1038/ncomms8440 (2015).

## Supplementary Material

Supplementary Information Supplementary Figures 1-8, Supplementary Table 1, Supplementary Note 1, Supplementary Methods and Supplementary References

Supplementary Data 1 Sequences of proteins tested in this study

Supplementary Data 2 Scores of off-target sites identified from literature

## Figures and Tables

**Table 1:** Target site guidelines for TALE design.

**Target site guideline**	**Rationale**
Target A runs	The NI repeat is more specific with NI as its N- or C- terminal neighbour
Avoid 3′ A, C or G	The NI, HD and NN repeats are less specific at the C-terminal end
Avoid T in first position	Both the 5′ T preference and the NG repeat are less specific if the first repeat is NG
Use the SIFTED web tool to identify off-targets	The web tool incorporates all context effects and can evaluate effective specificity in the genome.

SIFTED, Specificity Inference for TAL-Effector Design; TALE, transcription activator-like effector.

The observed context effects were used to create simple guidelines to incorporate when designing TALEs. However, we recommend using the SIFTED web tool to predict specificity and locate potential off-target sites when designing a TALE protein to target a genomic region.
