# Supplementary material for: Context influences on TALE–DNA binding revealed by quantitative profiling
Source: Nat Commun. 2015 Jun 11;6:7440. doi: 10.1038/ncomms8440 (PMC4467457; doi:10.1038/ncomms8440)
Supplement: Supplementary Information — Supplementary Figures 1-8, Supplementary Table 1, Supplementary Note 1, Supplementary Methods and Supplementary References [file ncomms8440-s1.pdf]

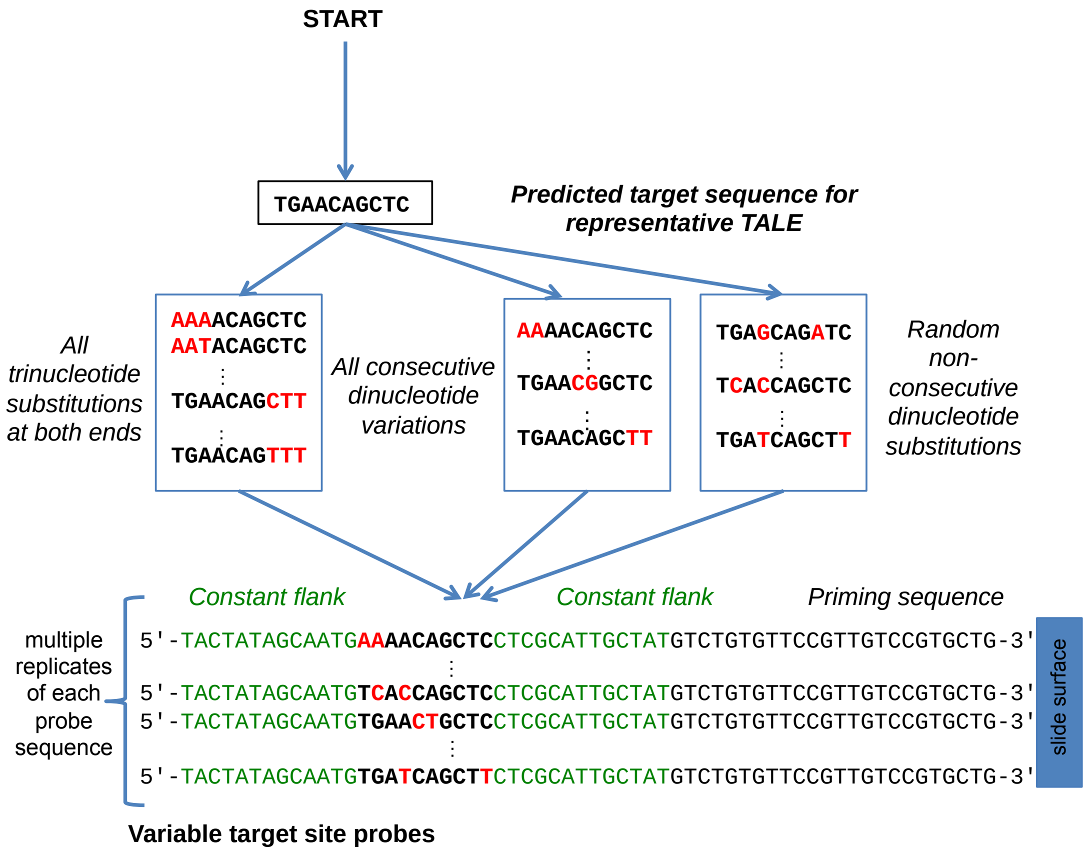

Supplementary Figure 1

Design of probes on custom arrays

Schematic representation of custom arrays design for a representative TALE protein. The distribution of probe sets on the 4 different custom array designs is described in Supplementary Note 1. Red font indicates variable sequences, while green font indicates constant sequence.

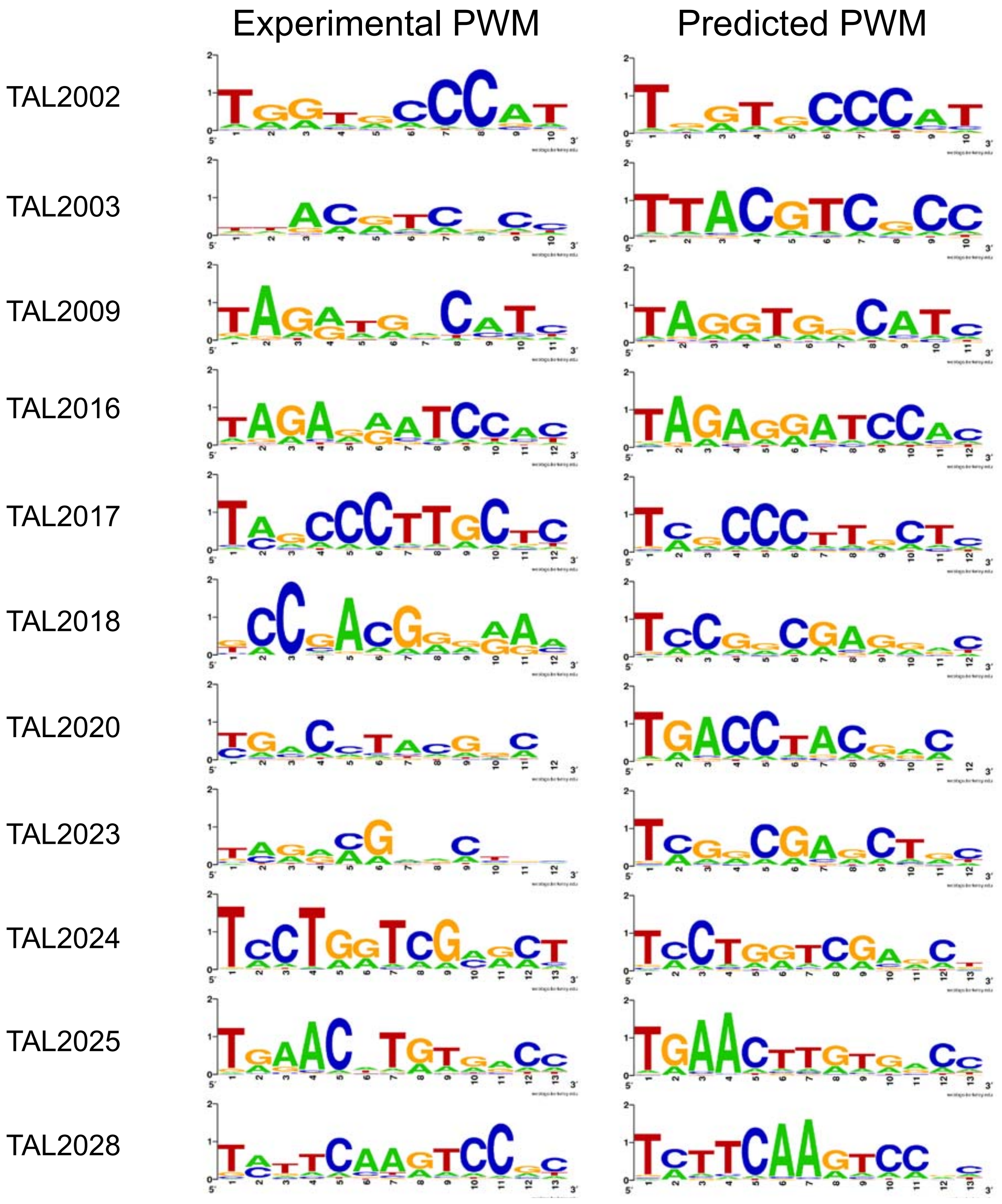
Experimental PWM
Predicted PWM
TAL2002
TAL2003
TAL2009
TAL2016
TAL2017
TAL2018
TAL2020
TAL2023
TAL2024
TAL2025
TAL2028

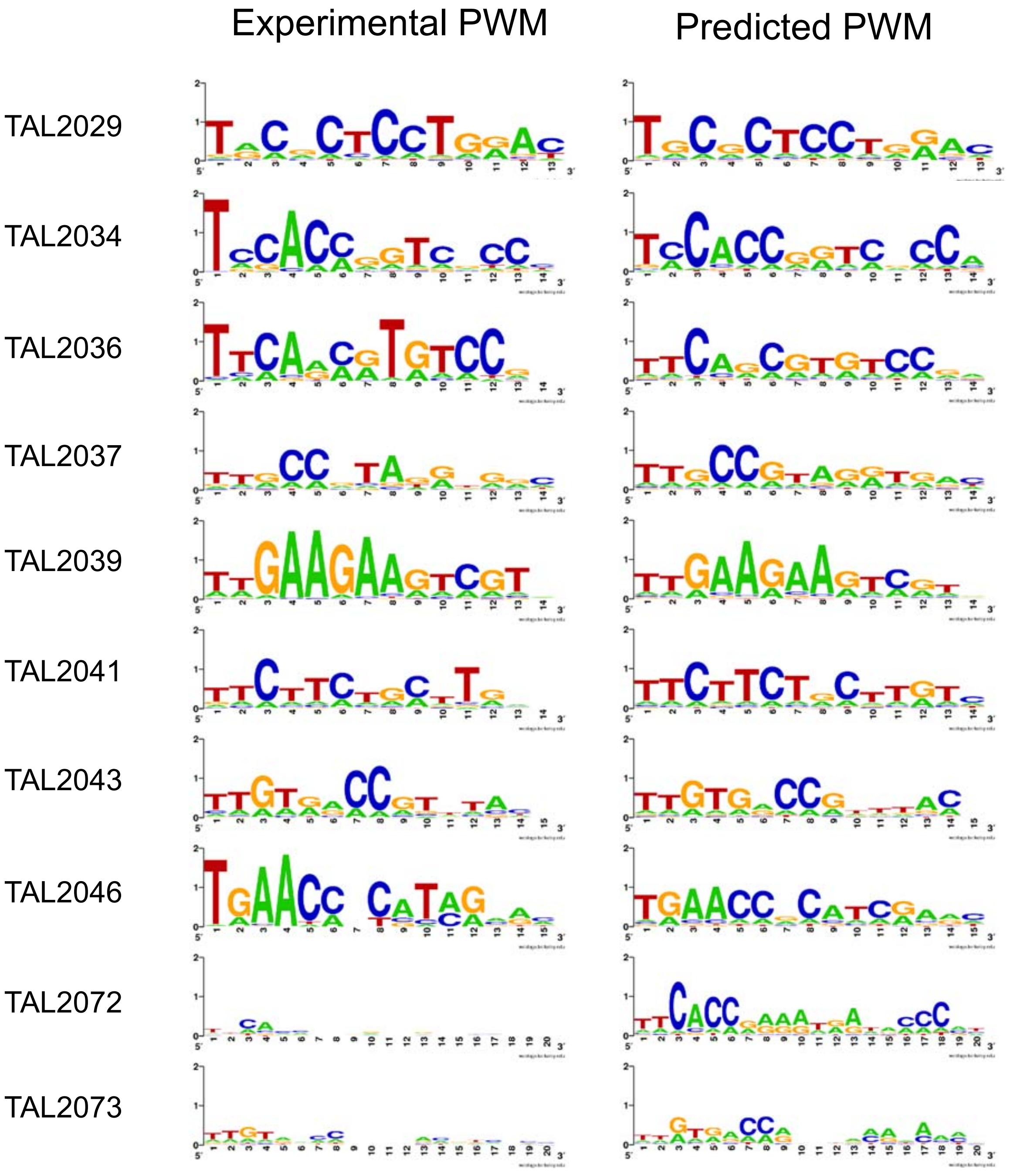

Supplementary Figure 2

Position weight matrices of all TALE proteins in this study

Experimental PWMs are derived from PBM experiments, as shown in Fig. 2a. Predicted PWMs are predicted by the SIFTED model, as described in Methods.

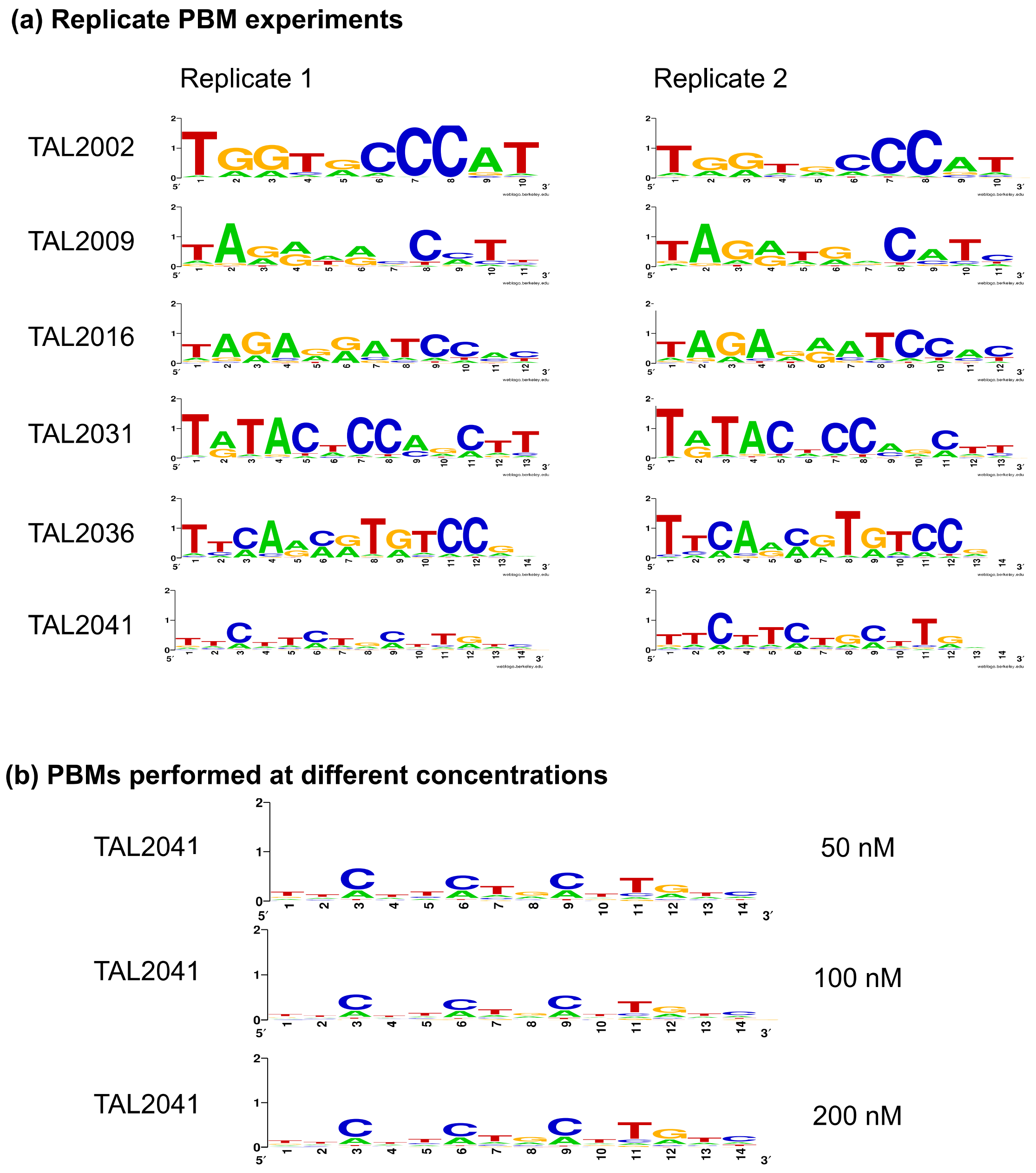

Supplementary Figure 3

PBM (a) replicates, or (b) experiments performed at different concentrations of the TALE protein.

(a) PWM logos are shown for two replicates of six TALE proteins. (b) PWMs are shown for one TALE protein, from PBM experiments performed at three concentrations. All PWMs are consistent across replicates and concentrations.

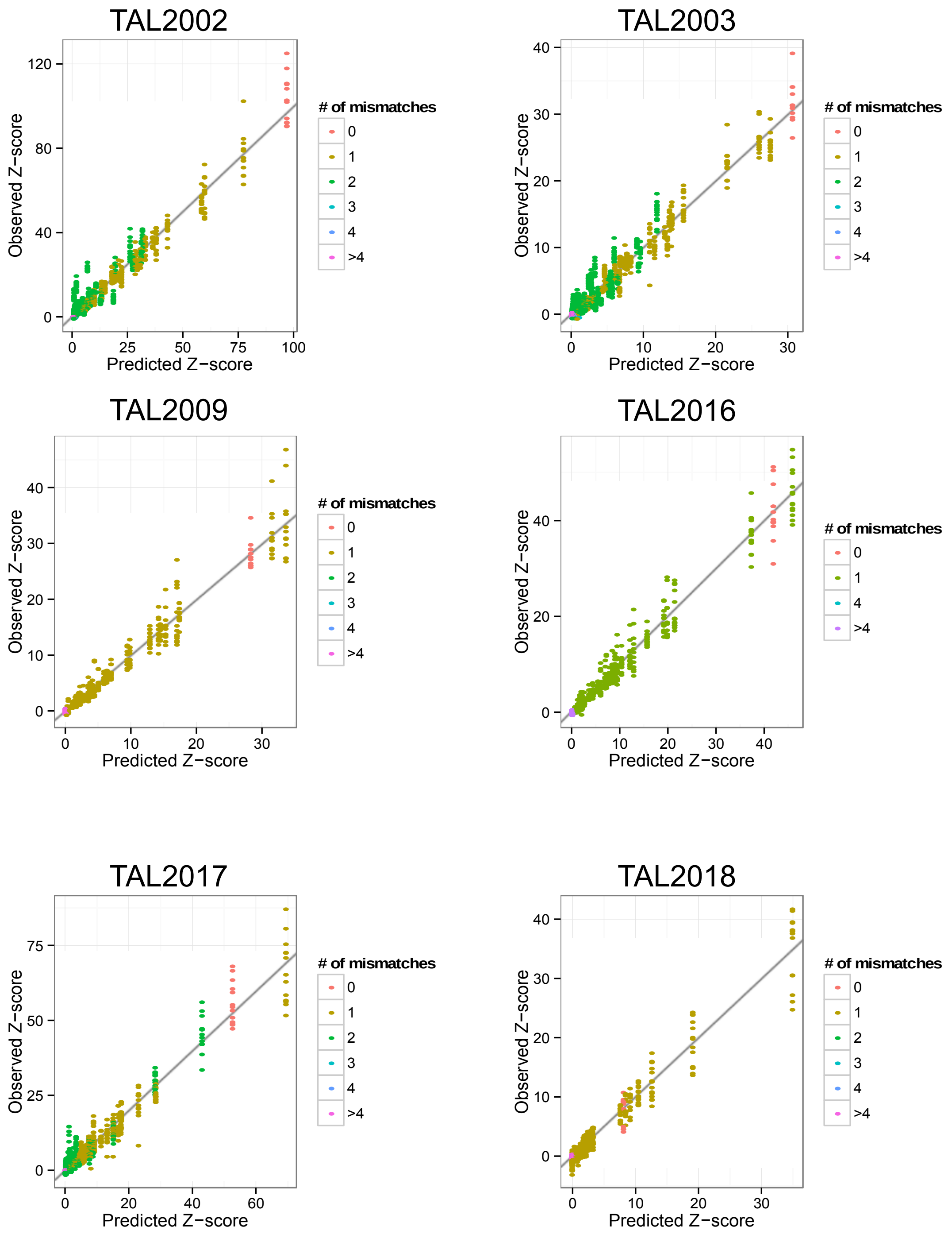
TAL2002
Observed Z−score
Predicted Z−score
0
40
80
120
0
25
50
75
100
# of mismatches
0
1
2
3
4
>4
TAL2003
Observed Z−score
Predicted Z−score
0
10
20
30
40
0
10
20
30
# of mismatches
0
1
2
3
4
>4
TAL2009
Observed Z−score
Predicted Z−score
0
10
20
30
40
0
10
20
30
# of mismatches
0
1
2
3
4
>4
TAL2016
Observed Z−score
Predicted Z−score
0
20
40
0
10
20
30
40
# of mismatches
0
1
4
>4
TAL2017
Observed Z−score
Predicted Z−score
0
25
50
75
0
20
40
60
# of mismatches
0
1
2
3
4
>4
TAL2018
Observed Z−score
Predicted Z−score
0
10
20
30
40
0
10
20
30
# of mismatches
0
1
2
3
4
>4

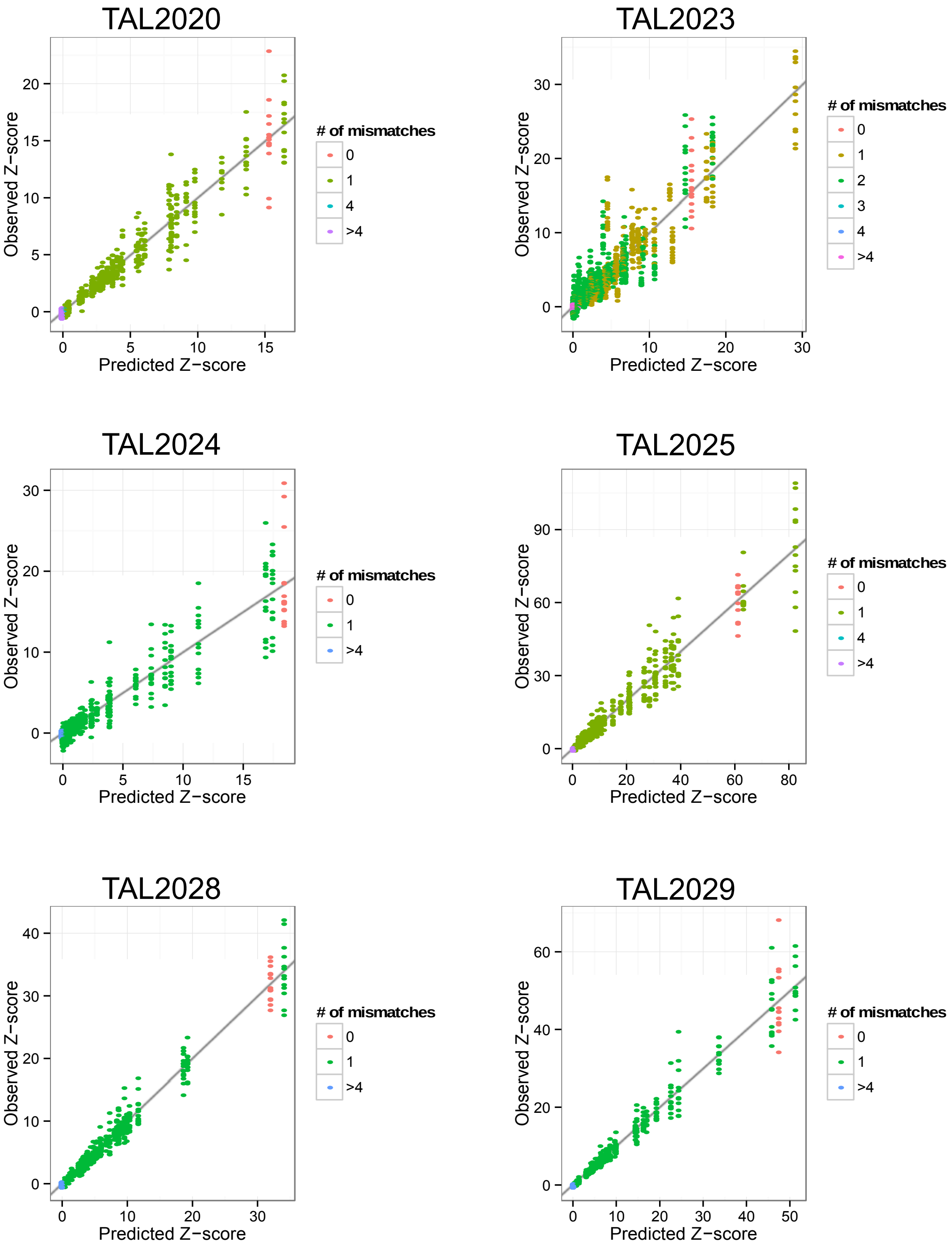
TAL2020
TAL2023
TAL2024
TAL2025
TAL2028
TAL2029
Observed Z−score
Predicted Z−score
# of mismatches
0
1
2
3
4
>4

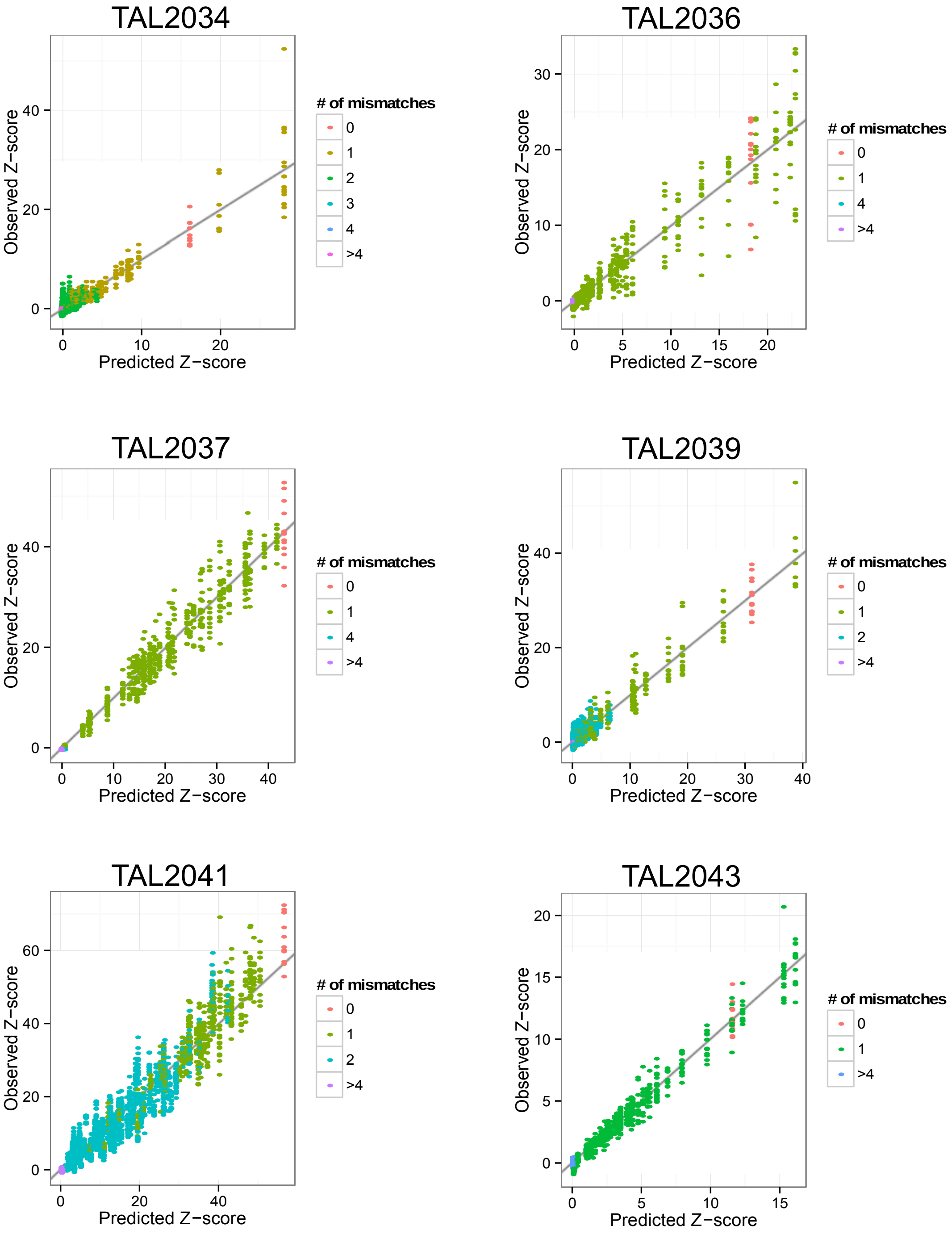
TAL2034
Observed Z−score
Predicted Z−score
# of mismatches
0
1
2
3
4
>4
TAL2036
Observed Z−score
Predicted Z−score
# of mismatches
0
1
4
>4
TAL2037
Observed Z−score
Predicted Z−score
# of mismatches
0
1
4
>4
TAL2039
Observed Z−score
Predicted Z−score
# of mismatches
0
1
2
>4
TAL2041
Observed Z−score
Predicted Z−score
# of mismatches
0
1
2
>4
TAL2043
Observed Z−score
Predicted Z−score
# of mismatches
0
1
>4

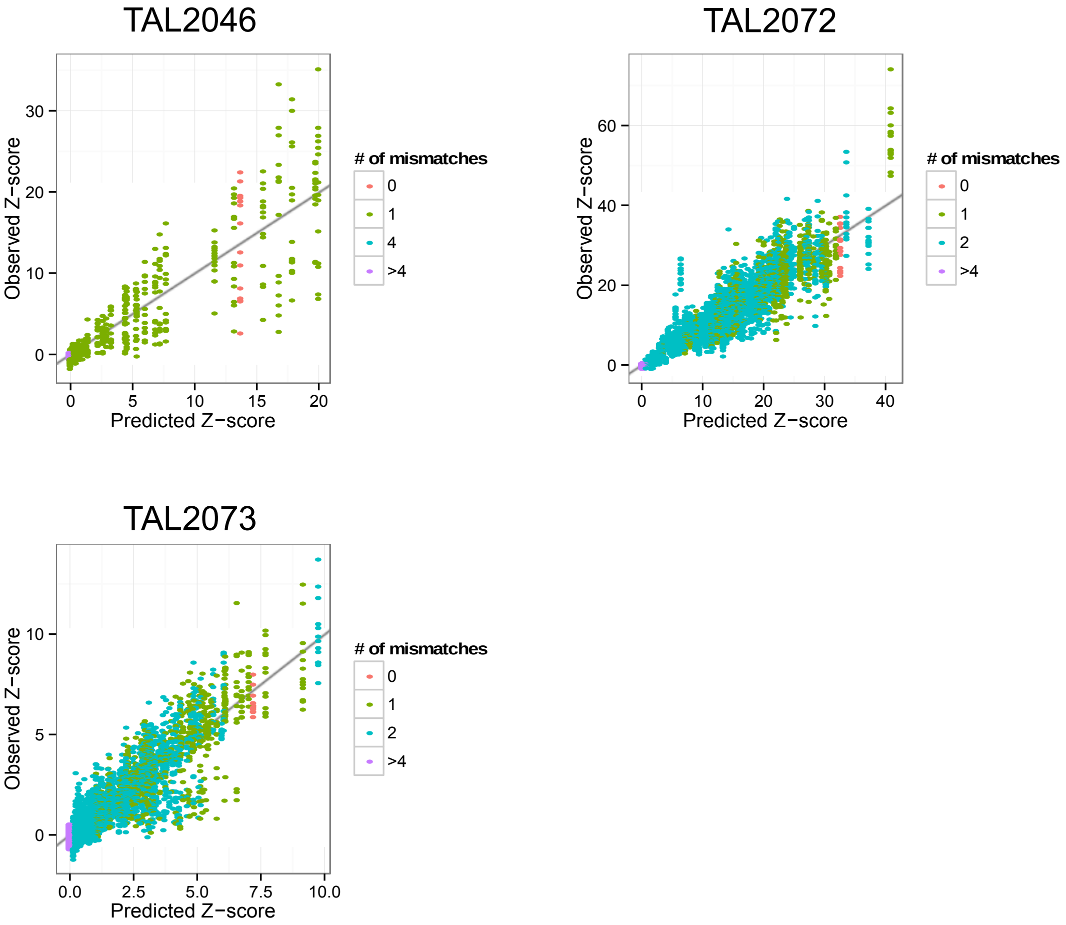

Supplementary Figure 4
Scatter plots of experimental vs. predicted probe intensity
Comparison between the probe z-scores measured by PBMs and the z-scores predicted by the derived PWM (see Methods). For each probe sequence, at least eight observed z-score replicates are shown. Points are colored by the number of mismatches between the sequence in the probe and the consensus sequence predicted from RVD identities as in Fig. 2b.

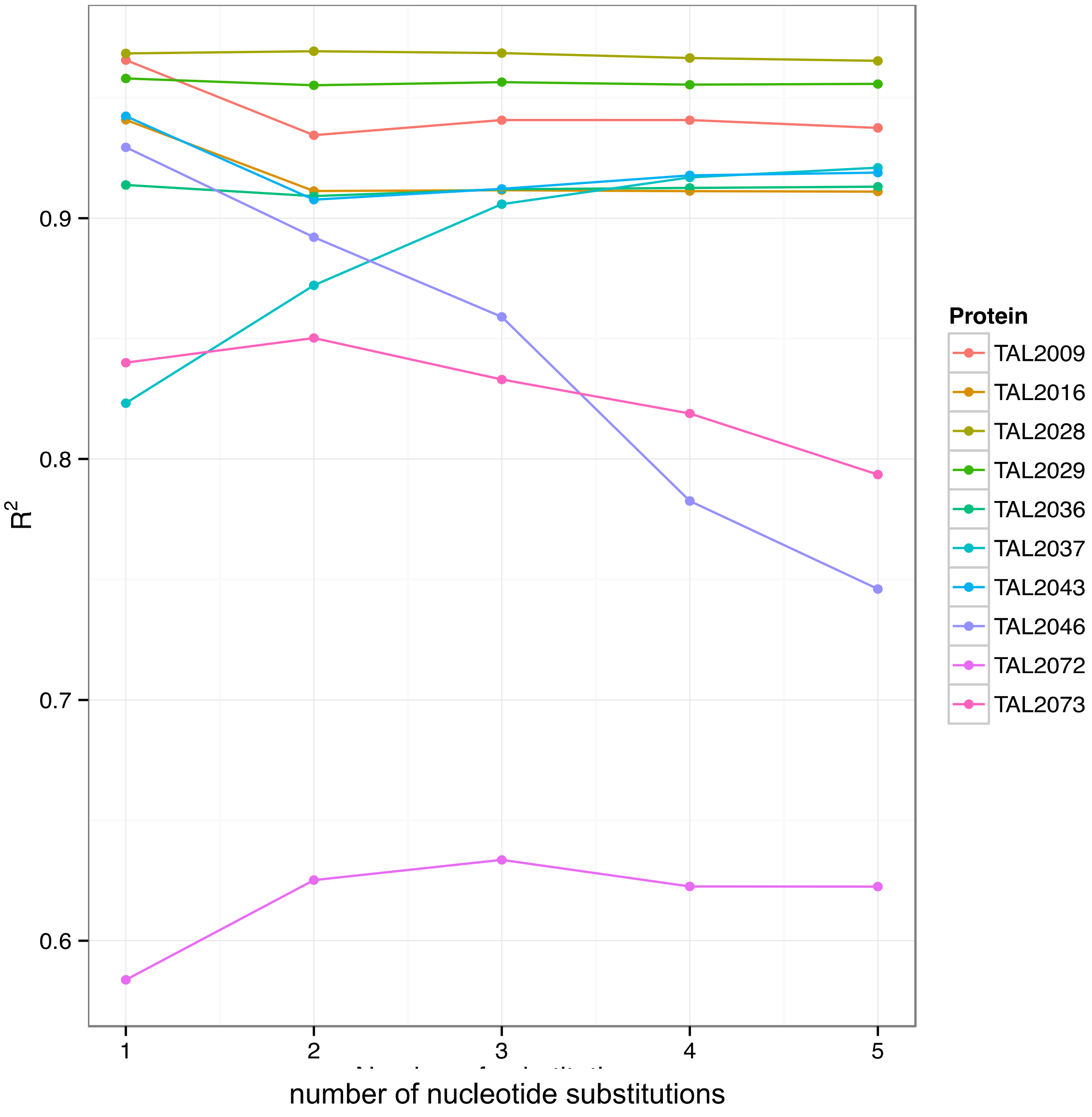

Supplementary Figure 5

PWMs can explain binding to probes with up to five nucleotide substitutions

R^2 between probe z-scores measured in PBMs and the z-score predicted by the derived PWM is shown for ten proteins. R^2 is defined as 1- (residual sum of squares) / (total sum of squares).

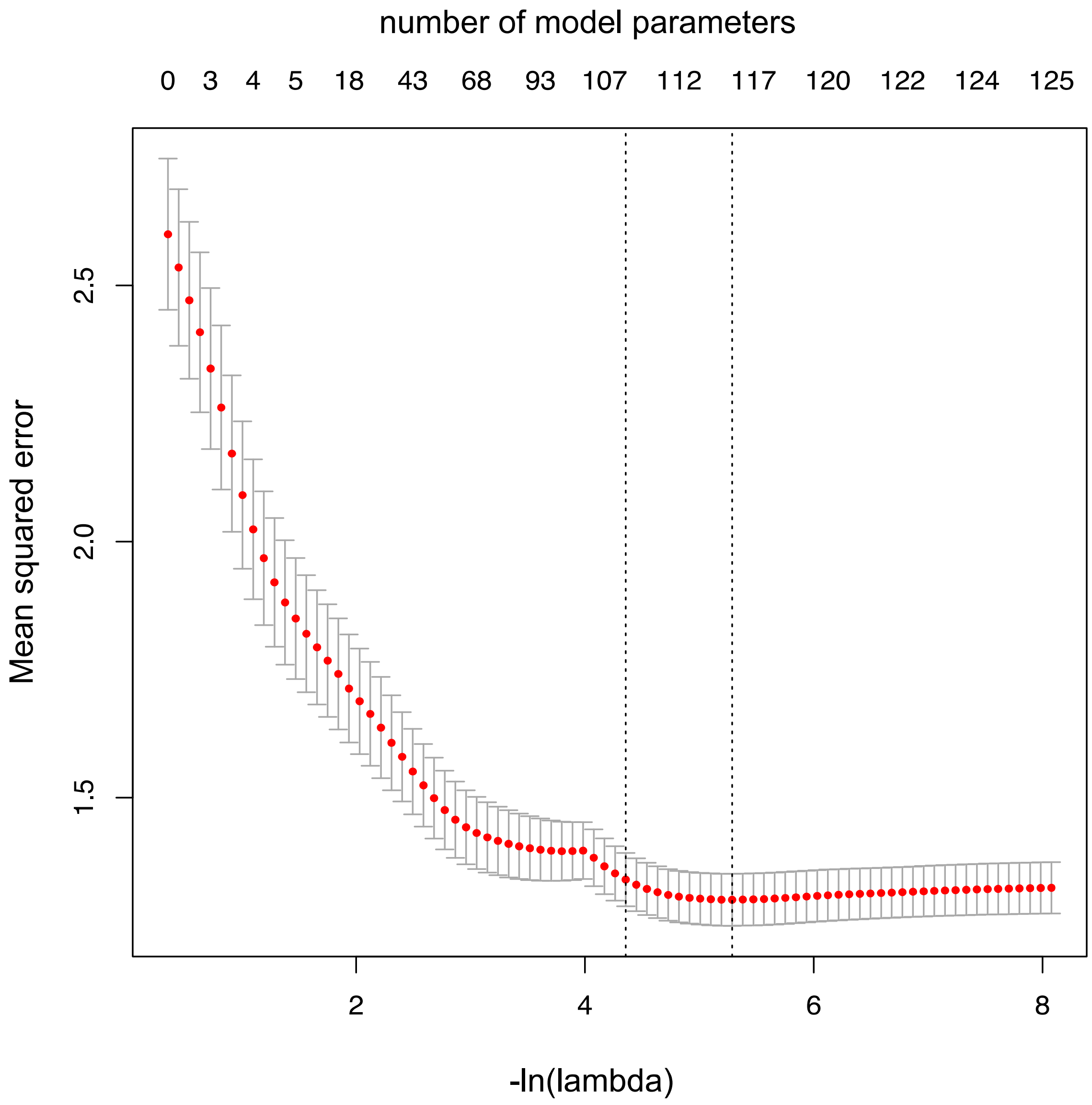

Supplementary Figure 6

SIFTED feature selection using Elastic Net Regularization

The mean squared error (MSE) is shown for different values of the regularization parameter lambda. Error bars represent 1 standard deviation. The right-most dashed vertical line shows the value of lambda with the lowest average MSE. The left-most dashed vertical line is the simplest model that performs within one standard deviation of the model with the lowest MSE, and was selected for the final model.

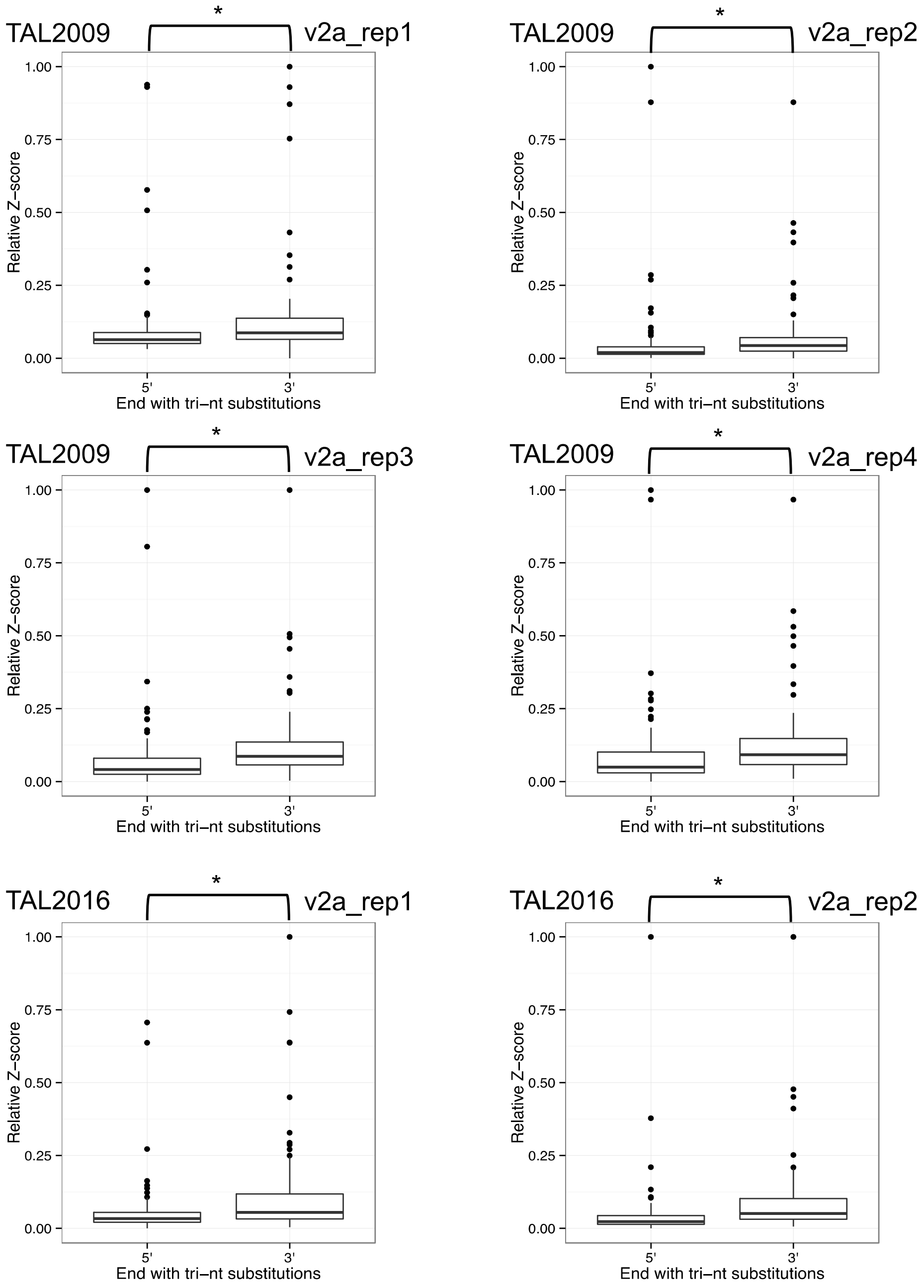
TAL2009
*
v2a_rep1
TAL2009
*
v2a_rep2
TAL2009
*
v2a_rep3
TAL2009
*
v2a_rep4
TAL2016
*
v2a_rep1
TAL2016
*
v2a_rep2
Relative Z−score
1.00
0.75
0.50
0.25
0.00
5'
3'
End with tri−nt substitutions

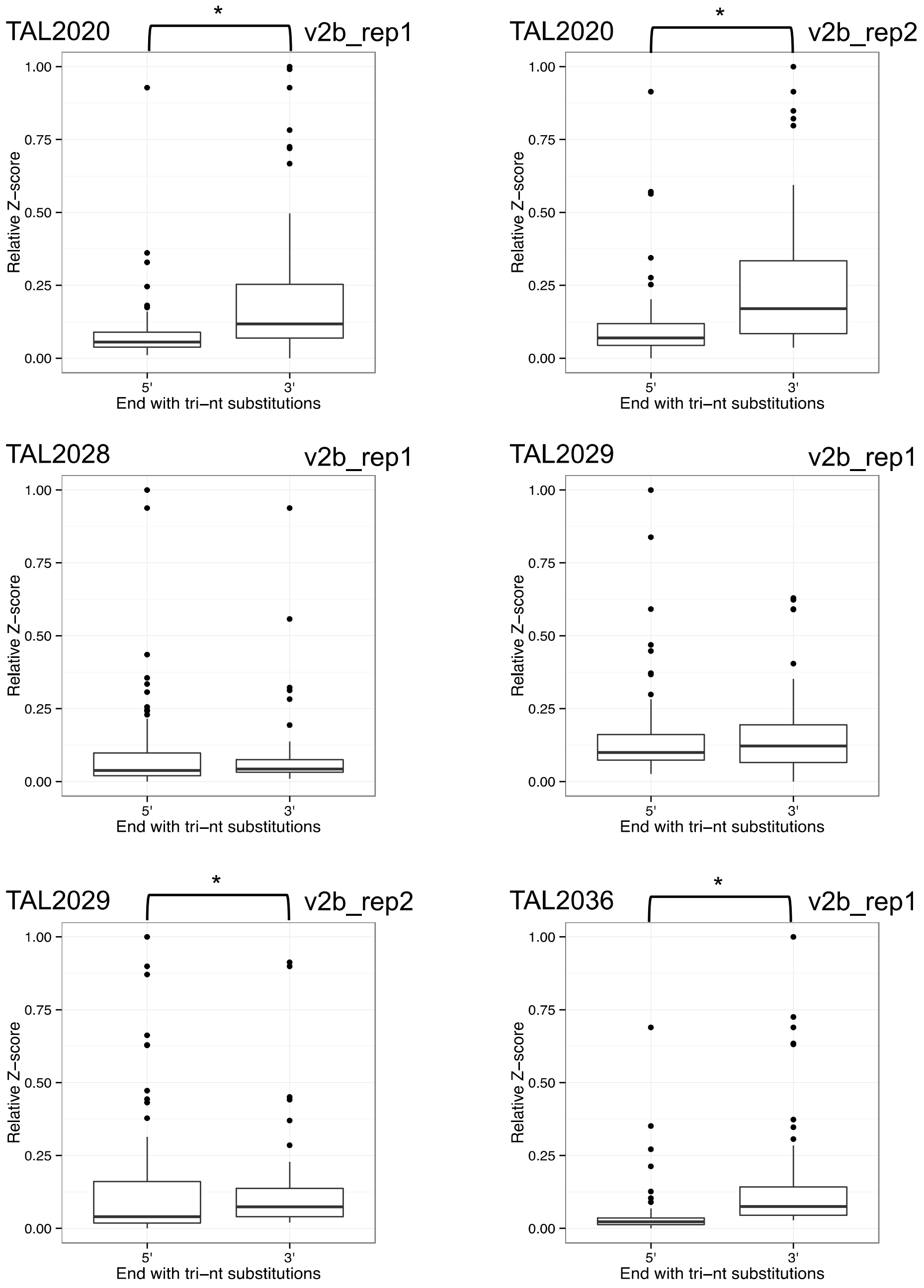

TAL2020
*
v2b_rep1
Relative Z−score
1.00
0.75
0.50
0.25
0.00
5'
3'
End with tri−nt substitutions
TAL2020
*
v2b_rep2
Relative Z−score
End with tri−nt substitutions
TAL2028
v2b_rep1
Relative Z−score
End with tri−nt substitutions
TAL2029
v2b_rep1
Relative Z−score
End with tri−nt substitutions
TAL2029
*
v2b_rep2
Relative Z−score
End with tri−nt substitutions
TAL2036
*
v2b_rep1
Relative Z−score
End with tri−nt substitutions

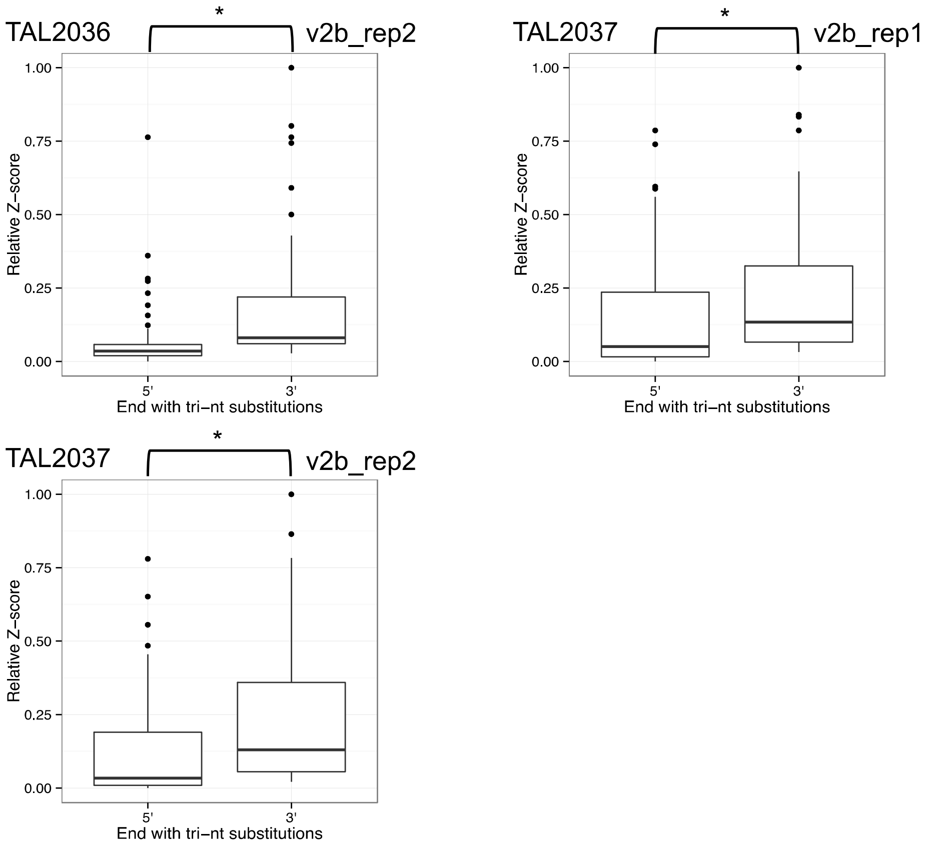

Supplementary Figure 7

Substitutions clustered at 5’ end of the TALE target site affect binding affinity more than those at the 3’ end

For each protein and experiment (named as in Supplementary Table 1), effects of substitutions clustered at the 5’ and 3’ end of the binding sites are shown. *Z*-scores are scaled such that the highest observed value is 1 and the lowest observed value is 0. Box plots are formatted as in Fig. 3a. * = $P < 0.05$, Wilcoxon signed-rank test.

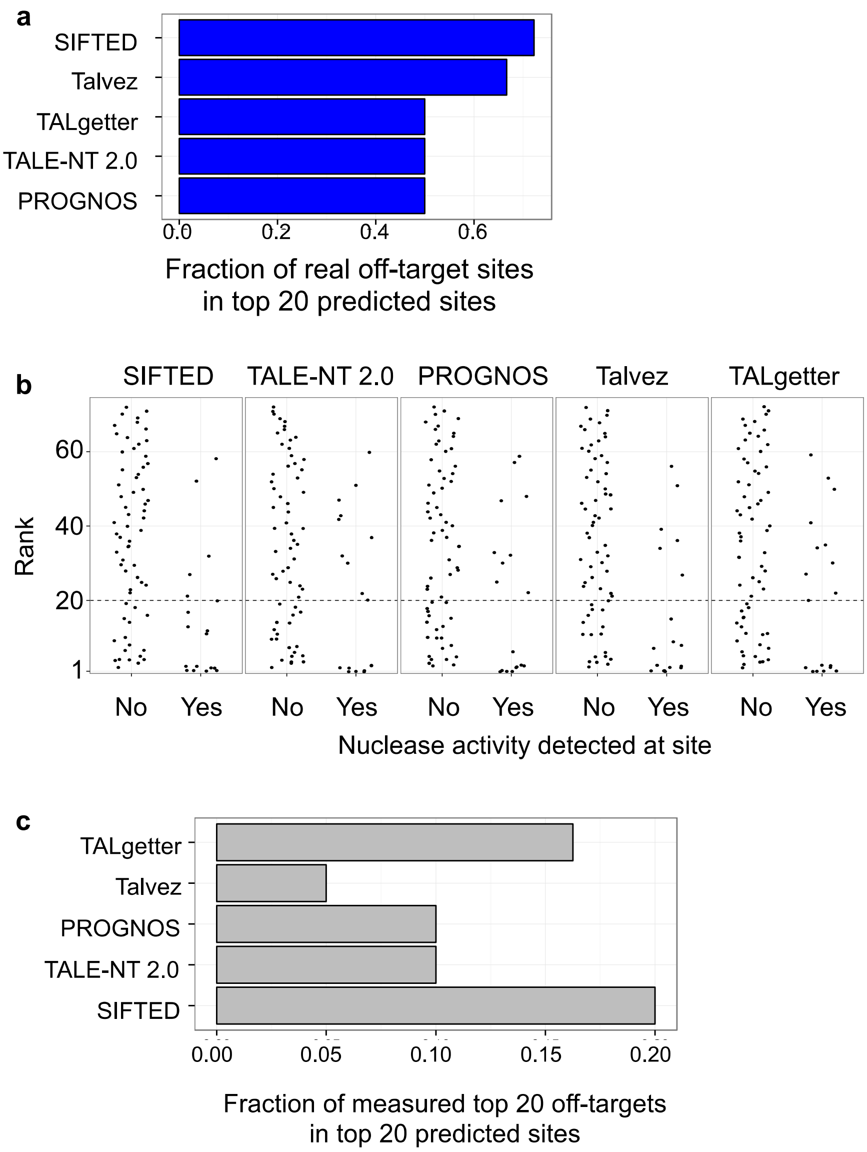

Supplementary Figure 8

(a) Each of the five tools was used to predict off-target sites for the TALEN pairs tested in Guilinger *et al*[1]. The fraction of the top twenty sites predicted by each of the tools that had reported nuclease activity is shown. **(b)** The distribution of ranks of target sites with and without reported nuclease activity in Guilinger *et al.* is shown. The rank of scores of the predicted sites, determined by each of the five tools, is shown on the y-axis, and presence or absence of detected nuclease activity is shown on the x-axis. The dashed line marks the top twenty ranked sites. **(c)** The five tools were used to predict the specificity of the TALE activators tested in Mali *et al*[2]. The fraction of the top twenty sites with reported TALE activity is shown for each tool.

Supplementary Table 1: PBM experimental conditions for 21 TALE proteins assayed by PBMs

Protein Name	Experiment Name	Target Site	PBM Concen-tration (nM)	Array Version (AMADID)	PWM used in training SIFTED?
TAL2002	TAL2002_ v2a_rep1	TGGTGCCCAT	250	059773	
TAL2002	TAL2002_ v2a_rep2	TGGTGCCCAT	200	059773	yes
TAL2003	TAL2003_ v2a_rep1	TTACGTCGCC	250	059773	
TAL2003	TAL2003_ v2a_rep2	TTACGTCGCC	200	059773	yes
TAL2009	TAL2009_ v1_rep1	TAGGTGGCATC	200	043197	
TAL2009	TAL2009_ v2a_rep1	TAGGTGGCATC	250	059773	
TAL2009	TAL2009_ v2a_rep2	TAGGTGGCATC	250	059775	yes
TAL2009	TAL2009_ v2a_rep3	TAGGTGGCATC	500	059773	
TAL2009	TAL2009_ v2a_rep4	TAGGTGGCATC	200	059773	
TAL2009	TAL2009_ v3_rep1	TAGGTGGCATC	200	063202	
TAL2016	TAL2016_ v1_rep1	TAGAGGATCCAC	200	043197	
TAL2016	TAL2016_ v2a_rep1	TAGAGGATCCAC	250	059773	
TAL2016	TAL2016_ v2a_rep2	TAGAGGATCCAC	250	059775	yes
TAL2016	TAL2016_ v3_rep1	TAGAGGATCCAC	200	063202	
TAL2017	TAL2017_ v2a_rep1	TCGCCCTTGCTC	200	059773	yes
TAL2018	TAL2018_ v1_rep1	TCCGGCGAGGGC	200	043197	
TAL2018	TAL2018_ v2b_rep1	TCCGGCGAGGGC	200	060799	yes
TAL2020	TAL2020_ v1_rep1	TGACCTACGGCG	200	043197	
TAL2020	TAL2020_ v2b_rep1	TGACCTACGGCG	250	060799	
TAL2020	TAL2020_ v2b_rep2	TGACCTACGGCG	200	060799	yes

TAL2023	TAL2023_ v2b_rep1	TCGGCGAGCTGC	100	060799	yes
TAL2024	TAL2024_ v1_rep1	TCCTGGTCGAGCT	200	043197	
TAL2024	TAL2024_ v2b_rep1	TCCTGGTCGAGCT	250	060799	yes
TAL2025	TAL2025_ v1_rep1	TGAACTTGTGGCC	200	043197	
TAL2025	TAL2025_ v2a_rep1	TGAACTTGTGGCC	250	059773	yes
TAL2028	TAL2028_ v1_rep1	TCTTCAAGTCCGC	200	043197	
TAL2028	TAL2028_ v2b_rep1	TCTTCAAGTCCGC	200	060799	yes
TAL2028	TAL2028_ v3_rep1	TCTTCAAGTCCGC	200	063202	
TAL2029	TAL2029_ v1_rep1	TGCGCTCCTGGAC	200	043197	
TAL2029	TAL2029_ v2b_rep1	TGCGCTCCTGGAC	250	060799	
TAL2029	TAL2029_ v2b_rep2	TGCGCTCCTGGAC	200	060799	yes
TAL2029	TAL2029_ v3_rep1	TGCGCTCCTGGAC	200	063202	
TAL2034	TAL2034_ v1_rep1	TCCACCGGTCGCCA	200	043197	yes
TAL2036	TAL2036_ v2b_rep1	TTCAGCGTGTCCGG	310	060799	
TAL2036	TAL2036_ v2b_rep2	TTCAGCGTGTCCGG	250	060799	yes
TAL2036	TAL2036_ v3_rep1	TTCAGCGTGTCCGG	200	063202	
TAL2037	TAL2037_ v2b_rep1	TTGCCGTAGGTGGC	340	060799	
TAL2037	TAL2037_ v2b_rep2	TTGCCGTAGGTGGC	250	060799	yes
TAL2037	TAL2037_ v3_rep1	TTGCCGTAGGTGGC	200	063202	
TAL2039	TAL2039_ v2b_rep1	TTGAAGAAGTCGTG	350	060799	yes
TAL2039	TAL2039_ v2b_rep2	TTGAAGAAGTCGTG	250	060799	
TAL2041	TAL2041_ v2a_rep1	TTCTTCTGCTTGTC	250	059775	
TAL2041	TAL2041_ v2a_rep2	TTCTTCTGCTTGTC	200	059773	yes
TAL2041	TAL2041_ v2a_rep3	TTCTTCTGCTTGTC	200	059773	

TAL2041	TAL2041_ v2a_rep4	TTCTTCTGCTTGTC	100	059773	
TAL2041	TAL2041_ v2a_rep5	TTCTTCTGCTTGTC	50	059773	
TAL2043	TAL2043_ v2a_rep1	TTGTGGCCGTTTACG	250	059775	yes
TAL2043	TAL2043_ v3_rep1	TTGTGGCCGTTTACG	200	063202	
TAL2046	TAL2046_ v1_rep1	TGAACCGCATCGAGC	200	043197	
TAL2046	TAL2046_ v2b_rep1	TGAACCGCATCGAGC	250	060799	yes
TAL2046	TAL2046_ v3_rep1	TGAACCGCATCGAGC	200	063202	
TAL2072	TAL2072_ v2b_rep1	TTCACCGGGGTGGTGCC CAT	50	060799	yes
TAL2072	TAL2072_ v3_rep1	TTCACCGGGGTGGTGCC CAT	150	063202	
TAL2073	TAL2073_ v2b_rep1	TTGTGGCCGTTTACGTC GCC	100	060799	yes
TAL2073	TAL2073_ v3_rep1	TTGTGGCCGTTTACGTC GCC	175	063202	
TAL2073	TAL2073_ v3_rep2	TTGTGGCCGTTTACGTC GCC	200	063202	

Supplementary Table 1: PBM experimental conditions for 21 TALE proteins assayed by PBMs

For all 21 proteins, target site and experimental conditions (array version and protein concentration) are provided. “Target site” is the target site predicted using the canonical TALE code. “Protein Concentration” is the TALE protein concentration used in the PBM experiment. “AMADID #” is the Agilent AMADID number of the array design used in the PBM experiment. The “PWM used in training SIFTED?” column indicates whether the PWM derived from that experiment was used to train the SIFTED model. For each protein, the experiment that produced the PWM with the highest R^2 was chosen.

Supplementary Note 1 – Probe sets included in custom TALE PBM designs

All consecutive dinucleotide substitutions within the target site. For each protein, the target site is predicted using the canonical TALE code, where the NI RVD targets A, HD targets C, NN targets G, and NG targets T. All target sites are preceded on the 5' end by a T. Sequences with all consecutive dinucleotide substitutions are generated. These target sites are positioned within constant flanking sequence. Present on array versions v1, v2a, and v2b.

Additional target site substitutions. The target site is predicted using the canonical TALE code, as above, and random sets of up to five substitutions are made. These target sites are positioned within constant flanking sequence. Present on array version v3.

Clusters of substitutions at 5' and 3' end of binding site. The target site is predicted using the canonical TALE code, as above, and clusters of three substitutions are introduced in the first three positions of the target site or in the last three positions. These target sites are positioned within constant flanking sequence. Present on array versions v2a and v2b.

The Agilent Array AMADID numbers for the array designs are: Version 1, 043197; Version 2a1, 059773; Version 2a2, 059775; Version 2b, 060799; and Version 3, 063202..

SUPPLEMENTARY METHODS

Position weight matrix model fitting

The statistical model below relates normalized probe signal intensity to the binding energy of the TALE protein to the binding site sequence represented on the probe. As an intermediate step, binding energy, ΔΔG, predicts the occupancy of the TALE protein on its binding site. Occupancy is linearly related to probe signal intensity.

PBM probe signal $Y_j = a + \frac{b}{1 + e^{\sum_i \Delta\Delta G_{i,j} - \mu}} + \epsilon_j$

$\Delta\Delta G_{i,j} \sim Exp(\beta)$ Energy matrix parameters (nucleotide *j* at position *i*)

$a \sim U(-100.0, 100.0)$

$b \sim U(0, 1000.0)$

(both) Define scaling between occupancy and signal intensities

$\mu \sim U(-20.0, 20.0)$ Chemical potential

$\epsilon_i \sim \mathcal{N}(0, \sigma^2)$ Gaussian noise

Algorithmic Approach of SIFTED web tool

In step 1, SIFTED requires that the candidate DNA target sequences are within the length range specified by the user and begin with a T on the 5' end. In step 2, the repeat sequence of the TALE that is predicted to target each candidate target DNA sequence is returned to the user. Alternatively, a user can input a given RVD sequence, and proceed from step 3 to predict its' specificity. To enumerate all sequences in step 3, we first define a graph where each node represents a binding site sequence and edges exist between all nodes that are separated by one nucleotide mismatch. The root node for the search is the one with the lowest predicted change in free energy (ΔΔG) value, which is set to 0. The edge weights (also interpretable as the distance

between nodes) correspond to the change in free energy between sequences (ΔΔΔG), starting at the root node. Therefore, the sum of edge weights for any path within the network that begins at the root node must be nonnegative (cf. Dijkstra's algorithm[3]). In practice, this implies that no neighbors of a node with free energy that exceeds the threshold can themselves satisfy the threshold criterion (unless they are reachable through other nodes). This implies that we can efficiently search the graph in a breadth-first manner by only including the neighbors of nodes that have a ΔΔΔG below the specified threshold in the search. In each iteration *k,* the nodes with *k* mismatches from the root node are explored, which also allows us to explicitly limit the search by the number of mismatches. As implemented online, the algorithm returns all binding site sequences below a particular relative K_d (off-target K_d / target K_d) threshold (by default set to 10), which is converted to a ΔΔG threshold using $\Delta\Delta G = \ln(K_d)$.

Once the target site sequences have been enumerated, we use a short-read mapping algorithm to find all of their genomic instances. Each of the predicted target site sequences is treated as if it were a read from a 2nd generation DNA sequencing instrument. However, since the input is given as a FASTA file, the read quality values are not applicable. We used *bowtie* v0.12.7 with flags "--all" (find all alignments for each read) and "-v 0" (report only hits with 0 end-to-end mismatches)[4]. The SAM output of bowtie is converted to a BED file with relative K_d values as interval scores, which the user can readily visualize or filter. Finally, the SIFTED pipeline returns a report in which the candidate proteins are ranked by the number of off-targets and their affinity by summing $1/K_d$ for all the predicted targets of a given protein.

Supplementary References

1 Guilinger, J. P. *et al.* Broad specificity profiling of TALENs results in engineered nucleases with improved DNA-cleavage specificity. *Nat Methods* **11**, 429-435, (2014).

2 Mali, P. *et al.* CAS9 transcriptional activators for target specificity screening and paired nickases for cooperative genome engineering. *Nat Biotechnol* **31**, 833-838, (2013).

3 Dijkstra, E. W. A Note on Two Problems in Connexion with Graphs. *Numerische Mathematik* **1**, 269-271, (1959).

4 Langmead, B., Trapnell, C., Pop, M. & Salzberg, S. L. Ultrafast and memory-efficient alignment of short DNA sequences to the human genome. *Genome Biol* **10**, R25, (2009).
